# Supplementary material for: Chronic social defeat stress induces meningeal neutrophilia via type I interferon signaling in male mice
Source: Nat Commun. 2025 Sep 1;16:8153. doi: 10.1038/s41467-025-62840-5 (PMC12402070; doi:10.1038/s41467-025-62840-5)
Supplement: Supplementary file 1 — Supplemental Information [file 41467_2025_62840_MOESM1_ESM.pdf]

Chronic social defeat stress induces meningeal neutrophilia via type I interferon signaling in male mice.

Authors: Stacey L. Kigar[1,2,3*†], Mary-Ellen Lynall[3,4†], Allison E. DePuyt[1], Robert Atkinson[1], Virginia H. Sun[1], Joshua D. Samuels[1], Nicole E. Eassa[1], Chelsie N. Poffenberger[1], Michael L. Lehmann[1], Samuel J. Listwak[1], Ferenc Livak[5], Abdel G. Elkahloun[6], Menna R. Clatworthy[4,7], Edward T. Bullmore[3], Miles Herkenham[1]

Affiliations:
[1]National Institute of Mental Health, Bethesda, MD, USA.
[2]Department of Medicine, University of Cambridge, UK
[3]Department of Psychiatry, University of Cambridge, UK.
[4]Molecular Immunity Unit, University of Cambridge Department of Medicine, Cambridge, UK.
[5] Laboratory of Genome Integrity, Flow Cytometry Core, National Cancer Institute, Bethesda, MD, USA.
[6] Microarrays and Single-Cell Genomics, National Human Genome Research Institute, Bethesda, MD, USA.
[7]Cellular Genetics, Wellcome Sanger Institute, UK.
[†]Indicates equal contribution
[*]Corresponding author at sk2128@cam.ac.uk

	β	standard error	z value	95% CI	p value	p(fdr)
%meningeal NPs (iv-)	-4.60	1.81	-2.55	[-4.50, -0.85]	0.011	0.016
%meningeal NPs (iv+)	-4.74	2.06	-2.30	[-4.74, -0.65]	0.021	0.021
%blood NPs	-5.12	1.72	-2.98	[-4.47, -1.12]	0.0029	0.0087

***Table S1*: Association between tissue-specific neutrophil populations and urine scent marking (USM)-related anhedonia in wild type mice.** Statistics from mixed generalized linear regression modeling with maximum likelihood estimation (MLE). Variables fitted to model according to the following formula: USM (marking, yes or no) ~ neutrophil population + cohort, family = binomial. NPs = neutrophils. *$p < 0.05$, **$p < 0.01$. (n_{HC} = 16 mice, n_{CSD} = 17 mice. N=5 experiments). Accompanies **Figure 2.** Source data are provided as a Supplemental Source Data file.

	β	standard error	t value	95% CI	p value	p(fdr)
%meningeal NPs (iv-)	-0.62	2.89	-0.21	[-0.46, 0.47]	0.83	0.83
%meningeal NPs (iv+)	-8.49	3.83	-2.21	[-1.39, -0.07]	0.033	0.10
%blood NPs	-3.38	2.24	-1.5	[-0.63, 0.20]	0.14	0.22

***Table S2*: Association between tissue-specific neutrophil populations and social interaction (SI)-related anhedonia behavior in wild type mice.** Statistics from mixed linear modeling with ordinary least squares (OLS) regression. Variables fitted to model according to the following formula: social approaches ~ neutrophil population + cohort. NPs = neutrophils. *$p < 0.05$. (n_{HC} = 22 mice, n_{CSD} = 26 mice. N=10 experiments). Accompanies **Figure 2.** Source data are provided as a Supplemental Source Data file.

	β	standard error	t value	95% CI	p value	p(fdr)
%meningeal NPs (iv-)	-1.88	0.96	-1.96	[-0.76, 0.02]	0.059	0.059
%meningeal NPs (iv+)	-2.88	1.10	-2.61	[-1.06, -0.13]	0.014	0.020
%blood NPs	-2.59	0.74	-3.50	[-0.82, -0.24]	0.0018	0.0053

***Table S3*: Associations between tissue-specific neutrophil populations and open field (OF)-related anxiety-like behavior in wild type mice.** Statistics from mixed linear modeling with ordinary least squares (OLS) regression. Variables fitted to model according to the following formula: novel arena exploration ~ neutrophil population + cohort. NPs = neutrophils. *$p < 0.05$, **$p < 0.01$. (n_{HC} = 21 mice, n_{CSD} = 23 mice. N=9 experiments). Accompanies **Figure 2.** Source data are provided as a Supplemental Source Data file.

	β	standard error	t value	95% CI	p value	p(fdr)
%meningeal NPs (iv-)	-11.6	4.76	-2.45	[-0.98, -0.08]	0.022	0.057
%meningeal NPs (iv+)	-14.2	6.47	-2.20	[-1.26, -0.04]	0.038	0.057
%blood NPs	-3.47	4.58	-0.76	[-0.84, 0.40]	0.46	0.46

***Table S4*: Associations between tissue-specific neutrophil populations and light dark (LD)-related anxiety-like behavior in wild type mice.** Statistics from mixed linear modeling with ordinary least squares (OLS) regression. Variables fitted to model according to the following formula: crosses to light ~ neutrophil population + cohort. NPs = neutrophils. *$p < 0.05$. (n_{HC} = 15 mice, n_{CSD} = 17 mice. N=7 experiments). Accompanies **Figure 2.** Source data are provided as a Supplemental Source Data file.

	n1	n2	t	95% CI Low	95% CI High	adj p value
HC v 1	16	7	0.21	-1.33	0.00	0.95
HC v 2	16	7	0.30	-1.87	0.00	0.95
HC v 4	16	9	2.18	-12.59	0.00	0.10
HC v 8	16	5	2.38	-16.89	0.00	0.083
HC v 14	16	10	2.71	-15.12	0.00	0.046

***Table S5*: Exposure to chronic, but not acute, defeat increases meningeal neutrophil levels in wild type mice.** Holm-Šídák's multiple comparisons test following 1-way ANOVA, comparing HC with samples collected from animals given varying numbers of days of defeat. n1/2 refer to individual mice. Accompanies **Figure 3A.** CSD = chronic social defeat (14 days), HC = home cage. Source data are provided as a Supplemental Source Data file.

comparison	n1	n2	t	95% CI Low	95% CI High	adj p value
HC v 1	16	7	2.59	-30.81	0.01	0.018
HC v 2	16	7	4.72	-56.26	0.00	<0.0001
HC v 4	16	9	2.71	-29.72	0.00	0.018
HC v 8	16	5	5.66	-76.20	0.00	<0.0001
HC v 14	16	10	5.34	-56.60	0.00	<0.0001

***Table S6*: Exposure to acute and chronic defeat increases blood neutrophil levels in wild type mice.** Holm-Šídák's multiple comparisons test following 1-way ANOVA, comparing HC with samples collected from animals given varying numbers of days of defeat. n1/2 refer to individual mice. Accompanies **Figure 3A.** CSD = chronic social defeat (14 days), HC = home cage. Source data are provided as a Supplemental Source Data file.

	n1	n2	t	95% CI Low	95% CI High	adj p value
HC v CSD	16	10	2.82	-15.12	0.00	0.030
HC v CSD+4h	16	3	1.61	-13.45	0.00	0.218
HC v CSD+8h	16	5	2.45	-16.67	0.00	0.056
HC v CSD+16h	16	4	5.00	-37.12	0.00	<0.0001
HC v CSD+1d	16	4	3.28	-24.34	0.00	0.011
HC v CSD+7d	16	4	0.35	0.00	2.58	0.730

***Table S7*: Meningeal neutrophil levels remain elevated for at least 24 h following cessation of CSD.** Holm-Šídák’s multiple comparisons test following 1-way ANOVA, comparing HC with samples collected from animals given varying amounts of time to recover from CSD (14 days of stress). NB: HC and CSD mice are the same as those shown in **Tables S5-6**. n1/2 refer to individual mice. Accompanies **Figure 3B.** CSD = chronic social defeat (14 days), HC = home cage. Source data are provided as a Supplemental Source Data file.

	n1	n2	t	95% CI Low	95% CI High	adj p value
HC v CSD	16	10	7.42	-54.65	-0.01	<0.0001
HC v CSD+4h	16	3	3.00	-35.52	0.00	0.023
HC v CSD+8h	16	5	0.20	0.00	1.94	0.944
HC v CSD+16h	16	4	0.70	-7.32	0.00	0.868
HC v CSD+1d	16	4	2.13	-22.38	0.00	0.149
HC v CSD+7d	16	4	0.30	-3.19	0.00	0.944

***Table S8*: Blood neutrophil levels remain elevated for at least 4 h following cessation of CSD.** Holm-Šídák’s multiple comparisons test following 1-way ANOVA, comparing HC with samples collected from animals given varying amounts of time to recover from CSD (14 days of stress). NB: HC and CSD mice are the same as those shown in **Tables S5-6**. n1/2 refer to individual mice. Accompanies **Figure 3B.** CSD = chronic social defeat (14 days), HC = home cage. Source data are provided as a Supplemental Source Data file.

	n_{HC}	n_{CSD}	t	95% CI	adj p value
meninges iv^-	8	9	3.221	[-20.42, -0.2743]	0.0428
meninges iv^+	8	9	2.398	[-1.073, 0.1256]	0.168
skull iv^-	8	9	4.901	[-18.93, -3.898]	0.0032
tibia iv^-	8	9	7.377	[-21.31, -8.867]	<0.0001
blood	7	9	8.808	[-65.93, -29.51]	<0.0001
spleen	8	9	5.56	[-9.065, -2.277]	0.0019

***Table S9*: Tissue-wide increase in neutrophils following CSD.** Šídák’s multiple comparisons test following Mixed-effects analysis (REML), comparing HC with CSD in different tissue sample collected from the same animals. n values refer to individual mice. Accompanies **Figure 5A.** CSD = chronic social defeat, HC = home cage. Source data are provided as a Supplemental Source Data file.

	meninges iv$^-$	meninges iv$^+$	blood	skull iv^{--}	spleen	tibia iv$^-$
meninges iv$^-$	-	0.508	0.498	0.832	0.570	0.747
meninges iv$^+$	0.56	-	0.716	0.456	0.605	0.443
blood	0.63	0.018	-	0.434	0.699	0.529
skull iv$^-$	0.00052	0.99	1.00	-	0.698	0.865
spleen	0.25	0.15	0.027	0.028	-	0.727
tibia iv$^-$	0.0085	1.00	0.44	0.00011	0.014	-

***Table S10*: Pearson correlations for neutrophil levels in different tissues.** Diagonal delineates p values and correlation coefficients. Bottom left triangle shows Bonferroni-corrected p values. Top right triangle shows correlation coefficients. Accompanies **Figure 5B.** See **Table S9** for n values**.** Source data are provided as a Supplemental Source Data file.

	n_{HC}	n_{CSD}	q	95% CI	adj p value
IgG:HC vs. IgG:CSD	8	8	3.95	[-15.67, -0.1646]	0.044
IgG:HC vs. IFNAR:HC	8	8	0.167	[-8.087, 7.417]	0.999
IgG:HC vs. IFNAR:CSD	8	7	0.768	[-9.617, 6.431]	0.948
IgG:CSD vs. IFNAR:HC	8	8	3.79	[-0.1704, 15.33]	0.057
IgG:CSD vs. IFNAR:CSD	8	7	3.05	[-1.700, 14.35]	0.161
IFNAR:HC vs. IFNAR:CSD	8	7	0.607	[-9.282, 6.766]	0.973

***Table S11*: Neutrophil elevation following CSD is halted with IFNAR antibody treatment in WT mice.** Tukey's multiple comparisons test following 2-way ANOVA. n values refer to individual mice. Accompanies **Figure 8F.** CSD = chronic social defeat, HC = home cage, IFNAR = interferon alpha type I receptor, IgG = immunoglobulin G. Source data are provided as a Supplemental Source Data file.

Wound score	Major criteria
1	No abrasions present
2-3	Abrasions
4	Abrasion + previous damage
5-6	Epidermal tears
7-8	Epidermal tears + previous damage
9-10	Laceration, deep tissue damage

Table S12: Criteria for assessing fight-related wounding.

Antibody-fluor:	Clone:	Vendor	Catalogue #
B220-BV711	RA3-6B2	Biolegend	103255
CD11b-APC/Fire750	M1/70	Biolegend	101262
CD11b-BUV737	M1/70	BD Biosciences	612800
CD11b-PE/Cy7	M1/70	Biolegend	101216
CD11b-PerCP/Cy5.5	M1/70	Biolegend	101228
CD11c-PE/Cy7	N418	Biolegend	117318
CD11c-PE/Dazzle594	N418	Biolegend	117348
CD19-BV711	6D5	Biolegend	115555
CD19-PE/Cy7	6D5	Biolegend	115520
CD206-BV650	C068C2	Biolegend	141723
CD3-AF488	17A2	Biolegend	100210
CD3-BV421	17A2	Biolegend	100228
CD3-PE	17A2	Biolegend	100206
CD3-PerCP/Cy5.5	17A2	Biolegend	100218
CD31-PE	390	Biolegend	102407
CD31-PE/Dazzle594	390	Biolegend	102430
CD4-BUV395	GK1.5	BD Biosciences	563790
CD4-BV421	RM4-5	Biolegend	100544
CD4-BV711	RM4-5	Biolegend	100550
CD44-APC	IM7	Biolegend	103012
CD44-BV785	IM7	Biolegend	103059
CD44-PE	IM7	Biolegend	103008
CD45-AF647	30-F11	Biolegend	103124
CD45-BUV395	30-F11	BD Biosciences	564279
CD45-BV421	30-F11	Biolegend	103134
CD45-FITC	30-F11	Biolegend	103108
CD62L-APC	MEL-14	Biolegend	104412
CD62L-BUV737	MEL-14	BD Biosciences	565213
CD8-BV421	53-6.7	Biolegend	100738
CD8-BV510	53-6.7	Biolegend	100751
CD8-PE/Cy7	53-6.7	Biolegend	100722
CXCR2-APC	SA044G4	Biolegend	149311
CXCR4-BV421	2B11	BD Biosciences	562738
ICAM-PE/Cy7	YN1/1.7.4	Biolegend	116121
IFNAR1-PE	MAR1-5A3	Biolegend	127312
Ly6C-BV650	HK1.4	Biolegend	128049
Ly6C-BV785	HK1.4	Biolegend	128041
Ly6C-PerCP/Cy5.5	HK1.4	Biolegend	128011
Ly6G-AF647	1A8	Biolegend	127610
Ly6G-APC	1A8	Biolegend	127614
Ly6G-BV510	1A8	Biolegend	127633
Ly6G-BV605	1A8	Biolegend	127639
Ly6G-PE/Cy7	1A8	Biolegend	127617
MHCI-BUV496	28-8-6	BD Biosciences	750133
MHCII-AF700	M5/114.15.2	ThermoFisher	56-5321-82
VCAM-APC	429 (MVCAM.A)	Biolegend	105717

Table S13: List of antibodies used for flow cytometry studies.

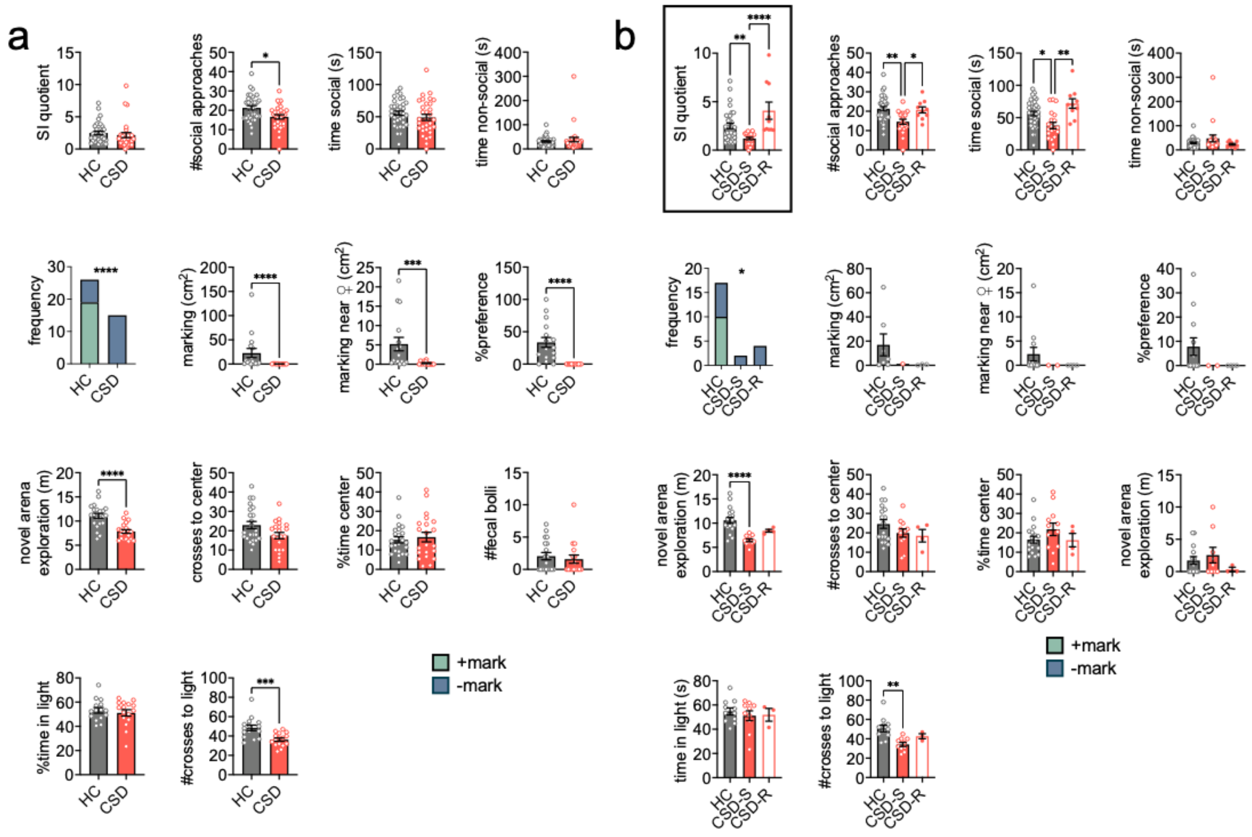

Figure S1: Behavioral testing in C57BL/6J mice following chronic social defeat (CSD) stress shows expected phenotype (NB: these animals were used for flow cytometry analyses shown in **Figures 1F-H & S3-4**). Behavioral testing began after 10 days of defeat stress; results are shown with all CSD animals combined (**a**), or with CSD animals stratified into susceptible vs resilient based on their performance in the social interaction (SI) test (**b**). *Top row*: For the SI test—which assesses social anhedonia as a depressive-like phenotype—SI quotient was calculated based on time spent engaging with the social stimulus divided by time spent engaging with the non-social stimulus (see **Figure 1C** for visualization). **a**) Mann Whitney test: p=0.075, U=378. n_{HC}=34, n_{CSD}=30. **b**) Kruskal-Wallis test: ****P<0.0001, H=21; Dunn's post-hoc: **$p_{HCvCSD\text{-}S}$=0.0013, $Z_{HCvCSD\text{-}S}$=3.5; ****$p_{CSD\text{-}SvCSD\text{-}R}$<0.0001, $Z_{CSD\text{-}SvCSD\text{-}R}$=4.2. n_{HC}=34, $n_{CSD\text{-}S}$=20, $n_{CSD\text{-}R}$=10. Social approaches represent the number of times the test mouse approached the social stimulus for sniffing. **a**) See **Figure 1C.** **b**) Kruskal-Wallis test: **P= 0.0023, H=12; Dunn's post-hoc: **$p_{HCvCSD\text{-}S}$=0.0025, $Z_{HCvCSD\text{-}S}$=3.3; *$p_{CSD\text{-}SvCSD\text{-}R}$=0.047, $Z_{CSD\text{-}SvCSD\text{-}R}$=2.4. n_{HC}=34, $n_{CSD\text{-}S}$=20, $n_{CSD\text{-}R}$=10. Time spent engaging with the social stimulus [in (**b**), Kruskal-Wallis test: ***P=0.0007, H=14.6; Dunn's post-hoc: *$p_{HCvCSD\text{-}S}$=0.012, $Z_{HCvCSD\text{-}S}$=2.9; **$p_{CSD\text{-}SvCSD\text{-}R}$=0.0012, $Z_{CSD\text{-}SvCSD\text{-}R}$=3.6. n_{HC}=34, $n_{CSD\text{-}S}$=20, $n_{CSD\text{-}R}$=10) and with the non-social stimulus, which comprise the SI quotient, are next. *Second row*: The urine scent marking (USM) task was used to assess sexual anhedonia, or depressive-like behavior; marking indicates engagement with a hedonic (i.e., female scent) stimulus. Data were stratified by task response–marking (+mark), or no marks present (-mark—see **Figure 1B. a**) Fisher's exact test, ****p<0.0001. n_{HC}=26, n_{CSD}=15. **b**) Fisher's exact test, *p=0.041. n_{HC}=17, $n_{CSD\text{-}S}$=2, $n_{CSD\text{-}R}$=4. Total area for test subject urine marking is shown [(**a**) Mann Whitney test: ****p<0.0001, U=14.5. n_{HC}=16, n_{CSD}=13], along with marks made in close proximity to the female scent [(**a**) Mann Whitney test: ***p=0.0005, U=29. n_{HC}=16, n_{CSD}=13], %Preference indicates preference for female scent, which is derived by dividing the marking area near the female urine spot by total marking area (**Figure 1B**). *Third*

row: The open field (OF) test was used to assess anxiety-like behavior (more willingness to explore the novel arena indicates less anxiousness). **a**) See **Figure 1D. b**) Kruskal-Wallis test: ****$P<0.0001$, $H=20$; Dunn's post-hoc: ****$p_{HCvCSD\text{-}S}<0.0001$, $Z_{HCvCSD\text{-}S}=4.5$. $n_{HC}=18$, $n_{CSD\text{-}S}=12$, $n_{CSD\text{-}R}=4$). We also tracked crosses into the center of the arena, time spent in the center of the arena, and number of fecal bolli as a crude proxy for stress, though these were not significant. *Bottom row:* The light/dark (LD) box assesses anxiety-like behavior—time spent exploring a brightly lit area as opposed to a covered dark space is quantified. Additionally, the number of crosses made from the dark side to the light side shows significant differences between groups: **a**) See **Figure 1E**. **b**) Kruskal-Wallis test: **$P=0.0033$, $H=11.4$; Dunn's post-hoc: **$p_{HCvCSD\text{-}S}=0.0022$, $Z_{HCvCSD\text{-}S}=3.4$. $n_{HC}=11$, $n_{CSD\text{-}S}=10$, $n_{CSD\text{-}R}=3$). Data points represent individual mice. All tests were two-tailed. Data shown as mean±SEM. Source data are provided as a Supplemental Source Data file.

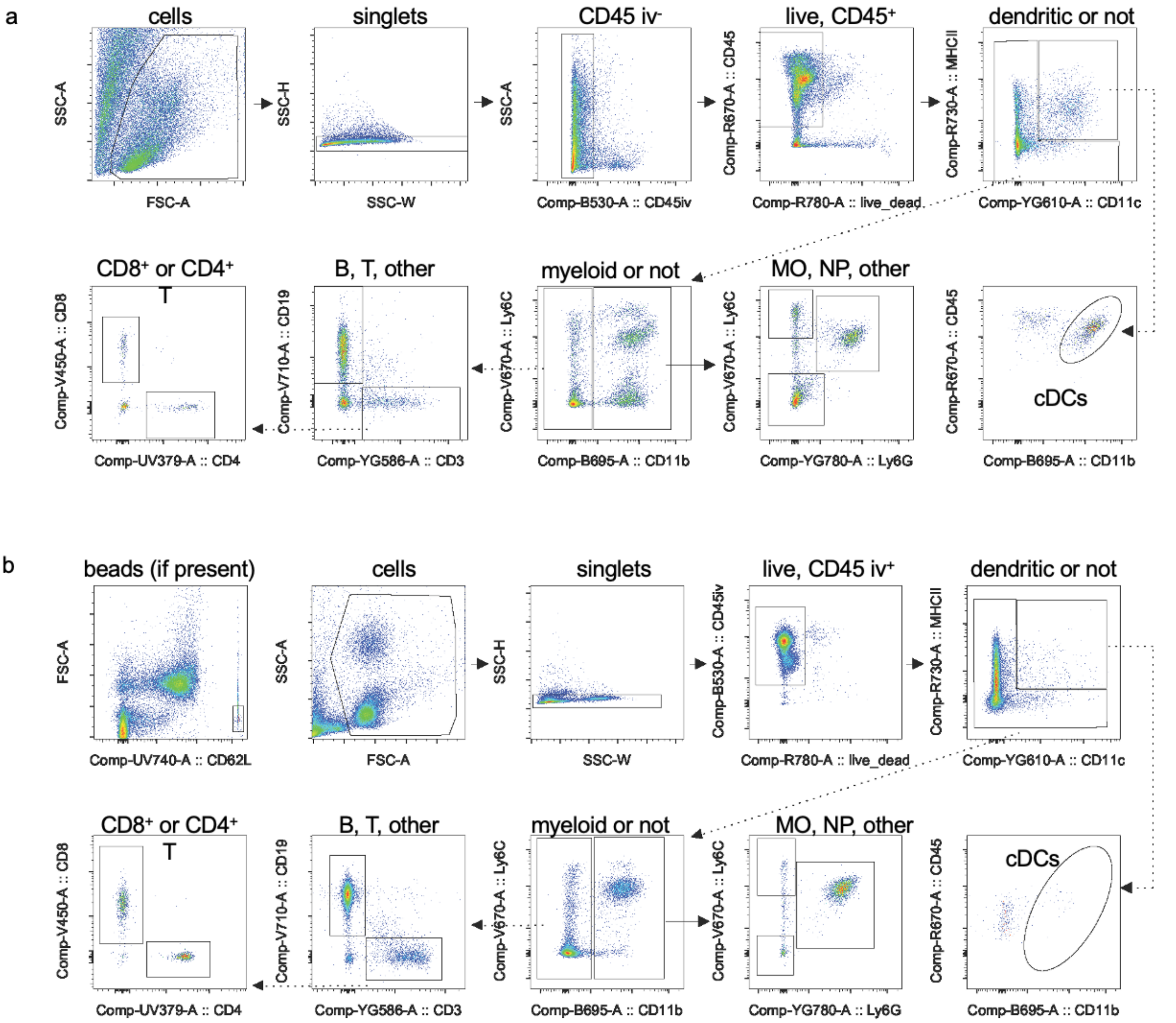

Figure S2: Full gating strategies for meninges and blood samples in C57BL/6J mice. **a**) Full gating strategy for meningeal tissue. Intravenous injection of a fluorescently labeled CD45 antibody prior to tissue harvest permitted differentiation of blood-exposed cells, e.g. due to imperfections in perfusion, fenestrated dural blood vessels permitting dye exposure, or persistent adherence to vascular lumen after perfusion. We assumed the resulting CD45iv^{-} population was enriched for leptomeningeal WBCs. To obtain absolute numbers of meningeal cells in a subset of samples, great care was taken to run the entire sample to completion. **b**) Full gating strategy for blood. To obtain absolute cell numbers in a subset of samples, CountBrite counting beads (ThermoFisher, catalog #C36950) were added just prior to data acquisition on a flow cytometer. HC = home cage, CSD = chronic social defeat stress, MO = monocyte, NP = neutrophil, cDCs = conventional dendritic cells.

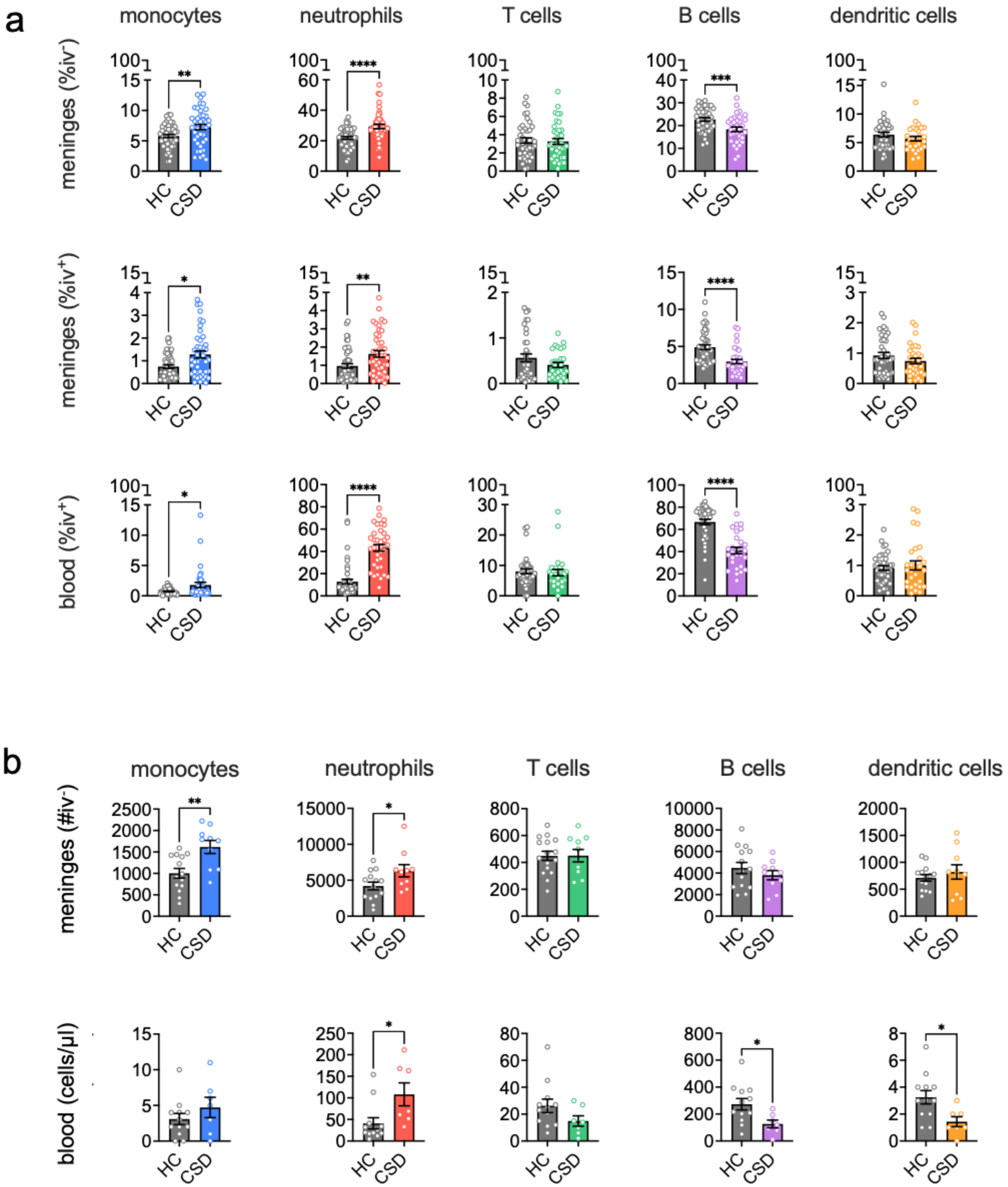

Figure S3: Chronic stress has multifactorial effects on immune cell subsets in different tissues. Note that these data are the same sample preparations presented in **Figure 1F-H**. Neutrophil data is shown to facilitate comparisons. **a**) *Top*: iv$^-$ meningeal tissue results (proportion relative to total live CD45$^+$ cells). CSD causes an increase in iv$^-$ meningeal monocytes (Mann Whitney test: $^{**}p$=0.0042, U=758, n_{HC}=52, n_{CSD}=44) and a decrease in iv$^-$ B cells (Unpaired t test: $^{***}p$=0.0009, t=3.5, df=77, n_{HC}=44, n_{CSD}=35). *Middle*: iv$^+$ meningeal tissue results (proportion

relative to total live $CD45^+$ cells). CSD causes an increase in iv^+ meningeal monocytes (Mann Whitney test: *p=0.012, U=803) and a decrease in iv^+ B cells (Unpaired t test: ****p<0.0001, t=4.3, df=77). *Bottom:* results from peripheral blood samples (proportion relative to total live $CD45^+$ cells). Monocyte levels are increased after CSD stress (Mann Whitney test: *p= 0.044, U=647, n_{HC}=47, n_{CSD}=37). Blood B cell levels are decreased after CSD stress (Unpaired t test: ****p<0.0001, t=6.7, df=65, n_{HC}=39, n_{CSD}=28); a subset of these animals was used to generate data reported previously[1]. **b**) For a subset of the samples in (**a**), absolute cell count information was available. *Top:* iv^- meningeal tissue results. Consistently with (**a**), iv^- meningeal monocyte levels were elevated (Unpaired t test: **p=0.0031, t=3.3, df=22, n_{HC}=14, n_{CSD}=10). *Bottom:* peripheral blood samples. While we did not detect an absolute decrease in meningeal B cells, we did detect the expected decrease in blood B cells (Unpaired t test: *p=0.026, t=2.4, df=17; n_{HC}=12, n_{CSD}=7). There was also a decrease in blood dendritic cells (Unpaired t test: *p=0.020, t=2.6, df=17; n_{HC}=12, n_{CSD}=7). Data points represent individual mice. All tests were two-tailed. HC = home cage, CSD = chronic social defeat stress. Data shown as mean±SEM. Source data are provided as a Supplemental Source Data file.

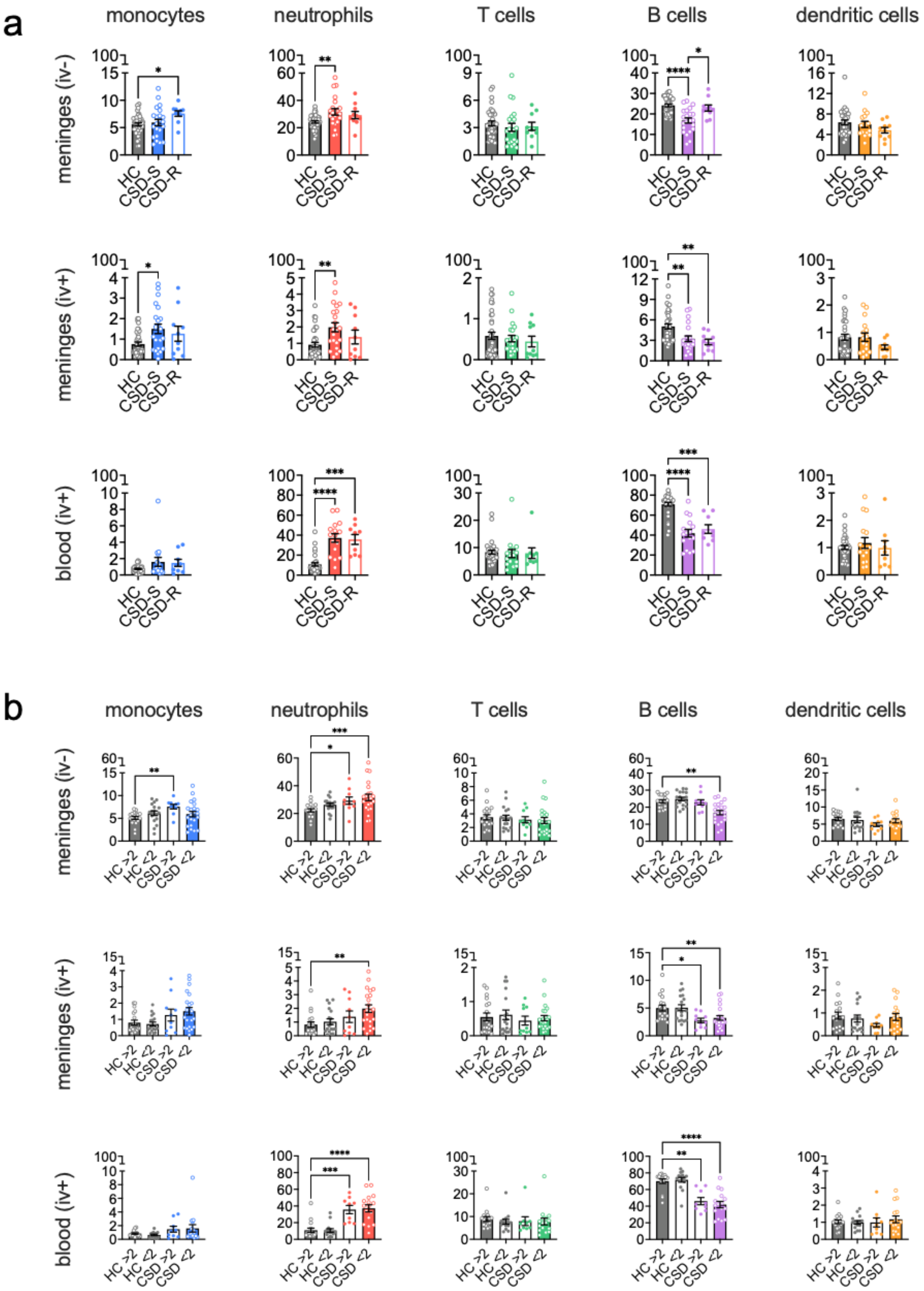
a
monocytes
neutrophils
T cells
B cells
dendritic cells
meninges (iv-)
meninges (iv+)
blood (iv+)
HC
CSD-S
CSD-R
b
monocytes
neutrophils
T cells
B cells
dendritic cells
meninges (iv-)
meninges (iv+)
blood (iv+)
HC >2
HC <2
CSD >2
CSD <2

Figure S4: Stratifying mice into ‘susceptible’ (CSD-S) vs ‘resilient’ (CSD-R) has no effect on flow cytometry results shown in **Figure S3**. **a**) *Top*: iv$^-$ meningeal tissue results (proportion relative to total live CD45$^+$ cells). Increased monocytes (Kruskal-Wallis test: *P=0.025, H=7.4; Dunn’s post-hoc: *$p_{\mathrm{HCvCSD\text{-}R}}$=0.023, $Z_{\mathrm{HCvCSD\text{-}R}}$=2.7). Increased neutrophils (Kruskal-Wallis test: *P=0.011, H=9.1; Dunn’s post-hoc: *$p_{\mathrm{HCvCSD\text{-}S}}$=0.015, $Z_{\mathrm{HCvCSD\text{-}S}}$=2.8). Reduced B cells (Kruskal-Wallis test: ****P<0.0001, H=24; Dunn’s post-hoc: ****$p_{\mathrm{HCvCSD\text{-}S}}$<0.0001, $Z_{\mathrm{HCvCSD\text{-}S}}$=4.8; *$p_{\mathrm{HCvCSD\text{-}R}}$=0.012, $Z_{\mathrm{HCvCSD\text{-}R}}$=2.9). n_{HC}=34, $n_{\mathrm{CSD\text{-}S}}$=20, $n_{\mathrm{CSD\text{-}R}}$=10. *Middle:* iv$^+$ meningeal tissue results (proportion relative to total live CD45$^+$ cells). Increased monocytes (Kruskal-Wallis test: *P=0.038, H=6.6; Dunn’s post-hoc: *$p_{\mathrm{HCvCSD\text{-}S}}$=0.035, $Z_{\mathrm{HCvCSD\text{-}S}}$=2.5). Increased neutrophils (Kruskal-Wallis test: *P=0.010, H=9.2; Dunn’s post-hoc: **$p_{\mathrm{HCvCSD\text{-}S}}$=0.0073, $Z_{\mathrm{HCvCSD\text{-}S}}$=3.0). Reduced B cells (Kruskal-Wallis test: ***P=0.0004, H=16; Dunn’s post-hoc: **$p_{\mathrm{HCvCSD\text{-}S}}$=0.0015, $Z_{\mathrm{HCvCSD\text{-}S}}$=3.5; *$p_{\mathrm{HCvCSD\text{-}R}}$=0.014, $Z_{\mathrm{HCvCSD\text{-}R}}$=2.8). n_{HC}=34, $n_{\mathrm{CSD\text{-}S}}$=20, $n_{\mathrm{CSD\text{-}R}}$=10. *Bottom:* peripheral blood results (proportion relative to total live CD45$^+$ cells). Increased neutrophils (Kruskal-Wallis test: ****P<0.0001, H=29; Dunn’s post-hoc: ****$p_{\mathrm{HCvCSD\text{-}S}}$<0.0001, $Z_{\mathrm{HCvCSD\text{-}S}}$=4.6; ***$p_{\mathrm{HCvCSD\text{-}R}}$=0.0003, $Z_{\mathrm{HCvCSD\text{-}R}}$=3.9). Reduced B cells (Kruskal-Wallis test: ****P<0.0001, H=30; Dunn’s post-hoc: ****$p_{\mathrm{HCvCSD\text{-}S}}$<0.0001, $Z_{\mathrm{HCvCSD\text{-}S}}$=4.9; ***$p_{\mathrm{HCvCSD\text{-}R}}$=0.0007, $Z_{\mathrm{HCvCSD\text{-}R}}$=3.7). n_{HC}=29, $n_{\mathrm{CSD\text{-}S}}$=14, $n_{\mathrm{CSD\text{-}R}}$=9. **b**) Additional stratification of HC animals into those with SI>2 or SI<2. *Top*: iv$^-$ meningeal tissue results (proportion relative to total live CD45$^+$ cells). Increased monocytes (Kruskal-Wallis test: *P=0.013, H=11; Dunn’s post-hoc: **$p_{\mathrm{HC>2vCSD>2}}$=0.0035, $Z_{\mathrm{HC>2vCSD>2}}$=3.2). Increased neutrophils (Kruskal-Wallis test: **P= 0.0018, H=15; Dunn’s post-hoc: *$p_{\mathrm{HC>2vCSD>2}}$=0.025, $Z_{\mathrm{HC>2vCSD>2}}$=2.6; ***$p_{\mathrm{HC>2vCSD<2}}$=0.0006, $Z_{\mathrm{HC>2vCSD<2}}$=3.7). Reduced B cells (Kruskal-Wallis test: ***P=0.0001, H=21; Dunn’s post-hoc: **$p_{\mathrm{HC>2vCSD<2}}$=0.0020, $Z_{\mathrm{HC>2vCSD<2}}$=3.4). $n_{\mathrm{HC>2}}$=18, $n_{\mathrm{HC<2}}$=18, $n_{\mathrm{CSD>2}}$=10, $n_{\mathrm{CSD<2}}$=22. *Middle:* iv$^+$ meningeal tissue results (proportion relative to total live CD45$^+$ cells). Increased neutrophils (Kruskal-Wallis test: *P=0.013, H=11; Dunn’s post-hoc: **$p_{\mathrm{HC>2vCSD<2}}$=0.0063, $Z_{\mathrm{HC>2vCSD<2}}$=3.1). Reduced B cells (Kruskal-Wallis test: ***P=0.0009, H=16; Dunn’s post-hoc: *$p_{\mathrm{HC>2vCSD>2}}$=0.017, $Z_{\mathrm{HC>2vCSD>2}}$=2.8; **$p_{\mathrm{HC>2vCSD<2}}$=0.0069, $Z_{\mathrm{HC>2vCSD<2}}$=3.0). $n_{\mathrm{HC>2}}$=18, $n_{\mathrm{HC<2}}$=18, $n_{\mathrm{CSD>2}}$=10, $n_{\mathrm{CSD<2}}$=22. *Bottom:* peripheral blood results (proportion relative to total live CD45$^+$ cells). Increased neutrophils (Kruskal-Wallis test: ****P<0.0001, H=30; Dunn’s post-hoc: ***$p_{\mathrm{HC>2vCSD>2}}$=0.0007, $Z_{\mathrm{HC>2vCSD>2}}$=3.7; ****$p_{\mathrm{HC>2vCSD<2}}$<0.0001, $Z_{\mathrm{HC>2vCSD<2}}$=4.3). Reduced B cells (Kruskal-Wallis test: ****P<0.0001, H=31; Dunn’s post-hoc: **$p_{\mathrm{HC>2vCSD>2}}$=0.0030, $Z_{\mathrm{HC>2vCSD>2}}$=3.3; ****$p_{\mathrm{HC>2vCSD<2}}$<0.0001, $Z_{\mathrm{HC>2vCSD<2}}$=4.2). $n_{\mathrm{HC>2}}$=16, $n_{\mathrm{HC<2}}$=15, $n_{\mathrm{CSD>2}}$=9, $n_{\mathrm{CSD<2}}$=16. Data shown as mean±SEM. Data points represent individual mice. HC = home cage, CSD = chronic social defeat stress. Source data are provided as a Supplemental Source Data file.

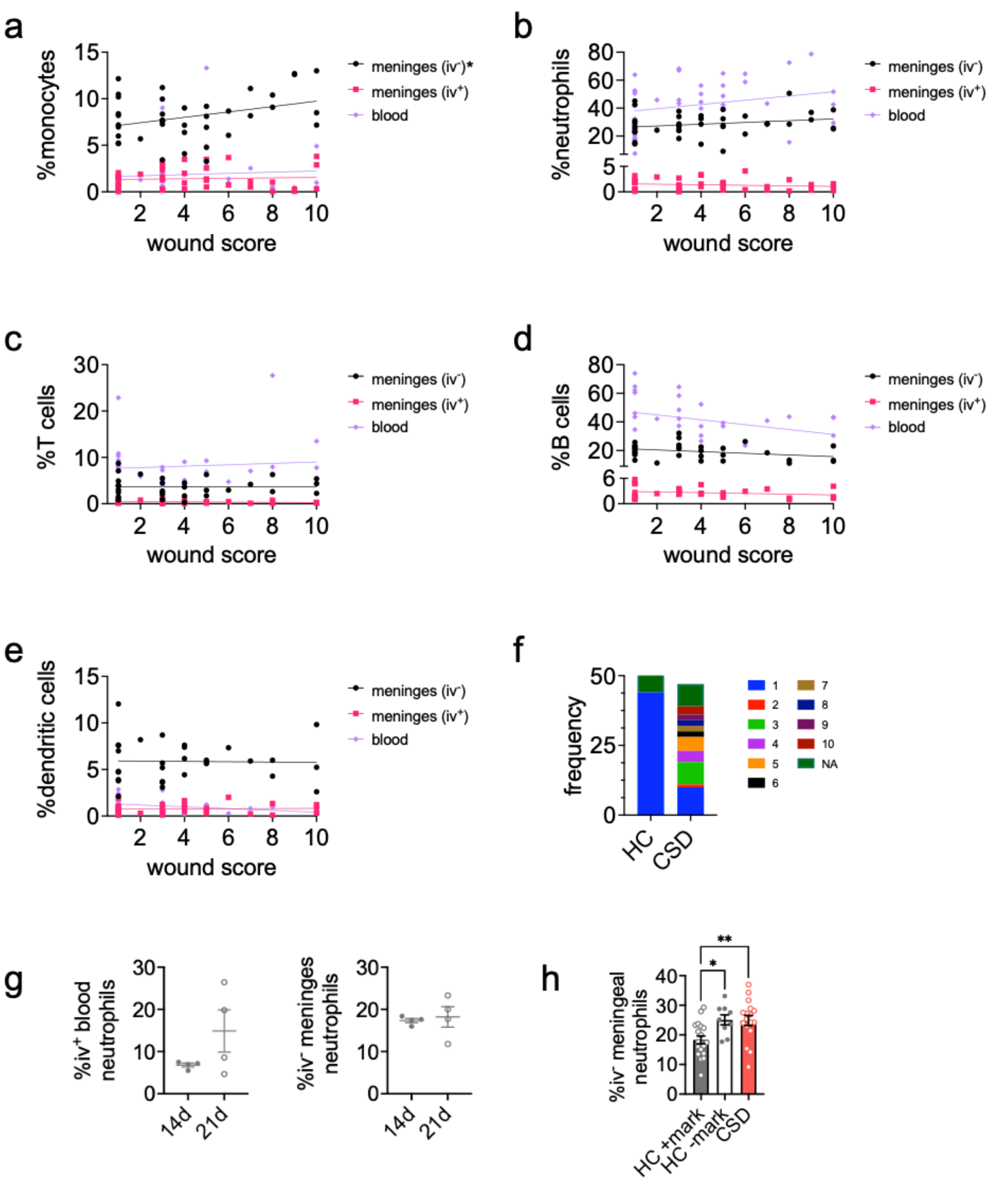

Figure S5: No relationships between neutrophils and wounding scores in CSD mice. However, wounding severity was associated with iv$^-$ meningeal monocyte levels. HC mice were excluded from this analysis as the distribution of their wound scores at 1 skews the analysis. **a**) *Monocytes*. iv$^-$ meningeal: β=0.30, Std error=0.14, **p*=0.034, n=37; iv$^+$ meningeal: β=0.0068, Std error=0.064, *p*=0.92, n=37; blood: β=0.054, Std error=0.15, *p*=0.73, n=36. **b**) *Neutrophils*. iv$^-$ meningeal: β=0.63, Std error=0.47, *p*=0.19, n=39; iv$^+$ meningeal: β=0.0068, Std error=0.47, *p*=0.37, n=39; blood: β=1.5, Std error=0.99, *p*=0.13, n=38. **c**) *T cells*. iv$^-$ meningeal: β=0.600,

Std error=0.13, p=0.98, n=30; iv^+ meningeal: β=-0.024, Std error=0.019, p=0.21, n=30; blood: β=0.15, Std error=0.36, p=0.68, n=29. **d**) *B cells*. iv^- meningeal: β=-0.59, Std error=0.33, p=0.090, n=30; iv^+ meningeal: β=-0.088, Std error=0.078, p=0.27, n=30; blood: β=-1.7, Std error=0.92, p=0.074, n=29. **e**) *Dendritic cells*. iv^- meningeal: β=-0.017, Std error=0.14, p=0.090, n=30; iv^+ meningeal: β=0.0089, Std error=0.034, p=0.79, n=30; blood: β=-0.096, Std error=0.047, p=0.051, n=29. **f**) Distribution of wound scores across both groups. **g**) Flow cytometry data from mice that arrived in our colony on the same day but were sacrificed a week apart (14 days post-delivery from Jackson Labs vs. 21 days). Critically, for iv- meningeal neutrophils there are no significant differences between mice housed in our facility for 14 days vs 21 days–though there is indeed more variation in blood neutrophils for HC mice at the 21 day time point, potentially underscoring the non-specific nature of the blood (compared to meningeal) neutrophils as a read-out for response (n = 4 per group; two-tailed tests run) **h**) HC mice that are 'anhedonic', i.e., do not show preference for female in the USM task, have elevated levels of neutrophils in the absence of other stressors (1-way ANOVA, **p=0.0040, $F_{(2,42)}$=6.31. Dunnett's post-hoc test: *$p_{HC+mark/HC-mark}$=0.019, $q_{HC+mark/HC-mark}$=2.7; **$p_{HC+mark/CSD}$=0.0055, $q_{HC+mark/CSD}$=3.2; $n_{HC+mark}$=19, $n_{HC-mark}$=9, n_{CSD}=17). Uncorrected p values for (**a-e**). Data points represent individual mice. HC = home cage, CSD = chronic social defeat stress, NA = not assessed. Data in (**g,h**) shown as mean±SEM. Source data are provided as a Supplemental Source Data file.

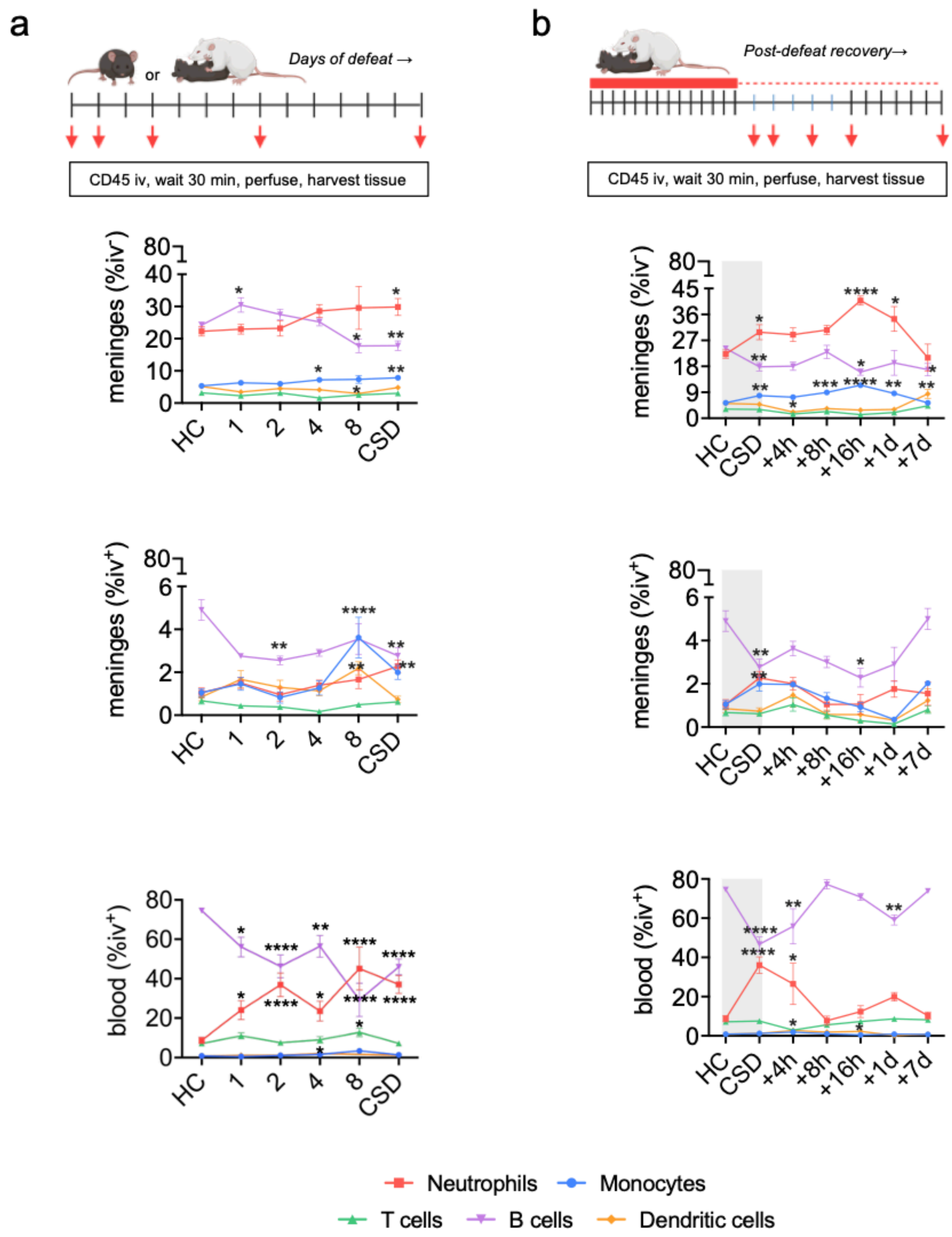

Figure S6: Chronic and acute stress, as well as recovery from CSD, cause tissue-specific immune cell fluctuations over time. Note that these data are the same sample preparations presented in **Figure 3**. **a)** *Top:* Schematic of acute vs chronic stress study; red arrows indicate time points in days at which mice were killed and tissue was harvested. *Bottom:* Visualization of different immune cell populations in iv$^-$ meninges, iv$^+$ meninges, and blood following increasing exposure to social defeat stress (subscript indicates days of defeat: n_{HC} = 8, n_1 = 7; n_2 = 7; n_4 = 9; n_8 = 4; n_{CSD} = 10). **b)** *Top:* Schematic of post-CSD recovery study; red arrows indicate time points in hours (blue ticks) or days (black ticks) at which mice were killed and tissue was

harvested. Gray shading indicates shared HC and CSD animals with (**a**), as experiments were done contemporaneously. *Bottom:* Visualization of different immune cell populations in iv^- meninges, iv^+ meninges, and blood for increasing intervals of CSD recovery (subscript indicates time post-CSD: $n_{4h} = 3$, $n_{8h} = 5$; $n_{16h} = 4$; $n_{1d} = 4$; $n_{7d} = 4$). See **Figure S7** for individual bar graphs and full statistics**.** HC = home cage, CSD = chronic social defeat. Data shown as mean ± SEM. *$p < 0.05$, **$p < 0.01$, ***$p < 0.001$, ****$p < 0.0001$. Source data are provided as a Supplemental Source Data file. Schematics created in BioRender. Kigar, S. (2025) https://BioRender.com/djwvueb.

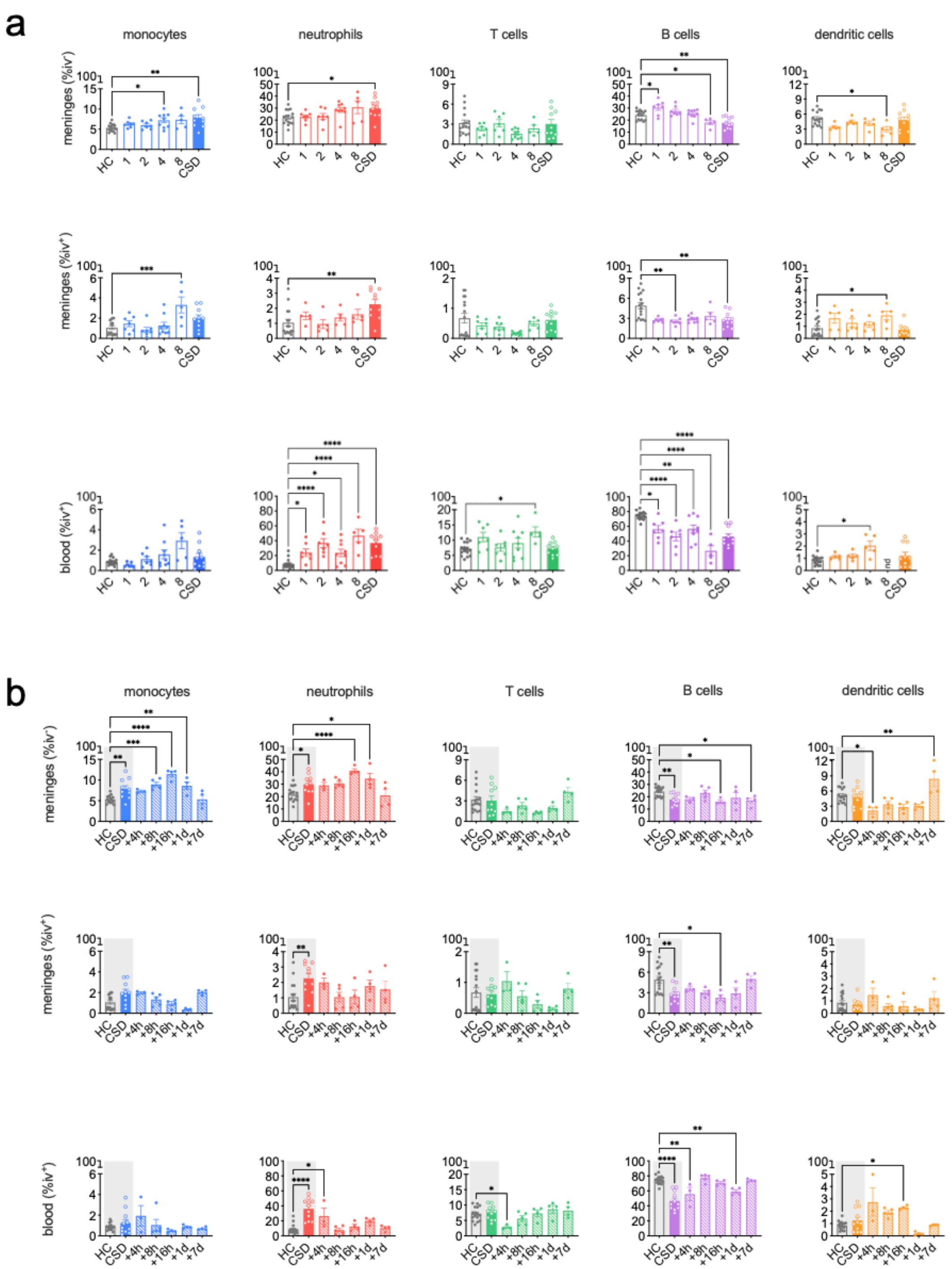

Figure S7**:** Chronic and acute stress act on immune cell subsets in a temporal- and tissue-specific manner***.*** Note that these data are the same sample preparations presented in **Figure 3** and **S6**. **a**) Like neutrophils, iv$^-$ monocytes accumulate in the meninges after 14 days of defeat stress, though they were also elevated after 4 days (Kruskal-Wallis test: *P=0.015, H=14.0; Dunn's test: *p_4=0.039, Z_4=2.7; **p_{14}=0.0068, Z_{14}=3.2). Likewise, iv$^+$ neutrophils were elevated after 14 days (1-way ANOVA, *p=0.017, $F_{(5,40)}$=3.2. Dunnett's test, q_{14}=3.7, **p_{14}<0.0033). Iv$^+$ monocytes were significantly elevated after 8 days, but not 14 (1-way ANOVA, ***p=0.0004, $F_{(5,48)}$=5.6. Dunnett's test, q_8=4.6, ***p_8=0.0001). For blood monocytes, there was a trend for

elevation at day 8 (Kruskal-Wallis test: *P=0.024, H=12.9; Dunn's test: p_8=0.08, Z_8=2.4). Iv$^-$ meningeal B cell levels first increased on day 1, then decreased by day 8 (1-way ANOVA: ****p<0.0001, $F_{(5,48)}$=10.1; Dunnett's test: *p_1=0.012, q_1=3.2; *p_8=0.033, q_8=2.8; **p_{14}=0.0032, q_{14}=3.6). Iv$^+$ meningeal B cells were decreased after 2 defeat encounters and remained decreased (Kruskal-Wallis test: **P=0.0047, H=16.9; Dunn's test: **p_2=0.0049, Z_2=3.3; **p_{14}=0.0086, Z_{14}=3.1). Blood B cell levels were reduced after the 1st defeat encounter (1-way ANOVA: ****P<0.0001, $F_{(5,48)}$=14.6; Dunnett's test: *p_1=0.010, q_1=3.2; ****p_2<0.0001, q_2=5.0; **p_4=0.0052, q_4=3.5; ****p_8<0.0001, q_8=7.4; ****p_{14}<0.0001, q_{14}=5.7). Additionally, there was a significant decrease in iv$^-$ meningeal DCs at day 8 (1-way ANOVA: *P=0.036, $F_{(5,40)}$=2.7; Dunnett's test: *p_8=0.019, Z_8=3.1). Blood T cell levels increased after 8 defeats (1-way ANOVA: *P=0.011, $F_{(5,48)}$=3.4; Dunnett's test: *p_8=0.010, Z_8=3.3). Conversely, iv$^+$ meningeal DCs increased at the same time point (1-way ANOVA: *P=0.017, $F_{(5,40)}$=3.1; Dunnett's test: *p_8=0.021, Z_8=3.0). No data are available for DCs in blood at day 8, but there was a trend for an increase in this population at day 4 (Kruskal-Wallis test: P=0.057, H=9.2; Dunn's test: *p_4=0.021, Z_4=2.8). **b**) Subscripts indicate hours post-defeat. *Top:* Like neutrophils, CSD meningeal iv$^-$ monocyte levels showed a main effect of time following stress cessation (One-way ANOVA: ****P<0.0001, $F_{(6,39)}$=11.2), remaining elevated for at least 24h (Dunnett's test: ***p_8=0.0004, q_8=4.4; ****p_{16}<0.0001, q_{16}=6.9; **p_{24}=0.0042, q_{24}=3.7). Iv$^-$ meningeal B cell levels also showed a main effect of time (One-way ANOVA: **P=0.0050, $F_{(6,39)}$=3.7), with reduced levels persisting for at least 7d (Dunnett's test: *p_{16}=0.018, q_{16}=3.2; *p_{168}=0.040, q_{168}=2.8). Iv$^-$ meningeal dendritic cells levels fluctuate over time (One-way ANOVA: ****P<0.0001, $F_{(6,39)}$=7.0), with reduced levels persisting for at least 7d (Dunnett's test: *p_4=0.033, q_4=2.9; **p_{168}=0.0050, q_{168}=3.6). *Middle*: Iv$^+$ meningeal monocytes and neutrophils both showed a main effect of time (Monocytes, Kruskal-Wallis test: **P=0.0016, H=21.3. Neutrophils, One-way ANOVA: *P=0.036, $F_{(6,39)}$=2.5), though neither showed statistically significant post-hoc differences. Iv$^+$ meningeal B cell levels were persistently reduced (One-way ANOVA: **P=0.0030, $F_{(6,39)}$=4.1) lasting at least 16h (Dunnett's test: *p_{16}=0.015, q_{16}=3.2). *Bottom:* There was a main effect of time post-CSD on blood T cell levels (One-way ANOVA: *P=0.026, $F_{(6,40)}$=2.7), dropping 4h into recovery (*p_4=0.023, q_4=3.0). Blood B cell levels were likewise reduced (One-way ANOVA: ****P<0.0001, $F_{(6,40)}$=16.7) up to 24h post-CSD (Dunnett's test: **p_4=0.0046, q_4=3.6; **p_{24}=0.0096, q_{24}=3.4). Blood dendritic cell levels fluctuate with time (Kruskal-Wallis test: ***P=0.0003, H=25.3), with elevated levels 16h post-CSD (Dunnett's test: *p_{16}=0.024, Z_{16}=2.9). Data points represent individual mice. Open bars indicate fewer defeat encounters than standard CSD paradigm, shaded bars indicate animals that underwent complete CSD paradigm and were given time to recover. HC=home cage, CSD=chronic social defeat stress, na=not assessed. Data shown as mean±SEM. Source data are provided as a Supplemental Source Data file.

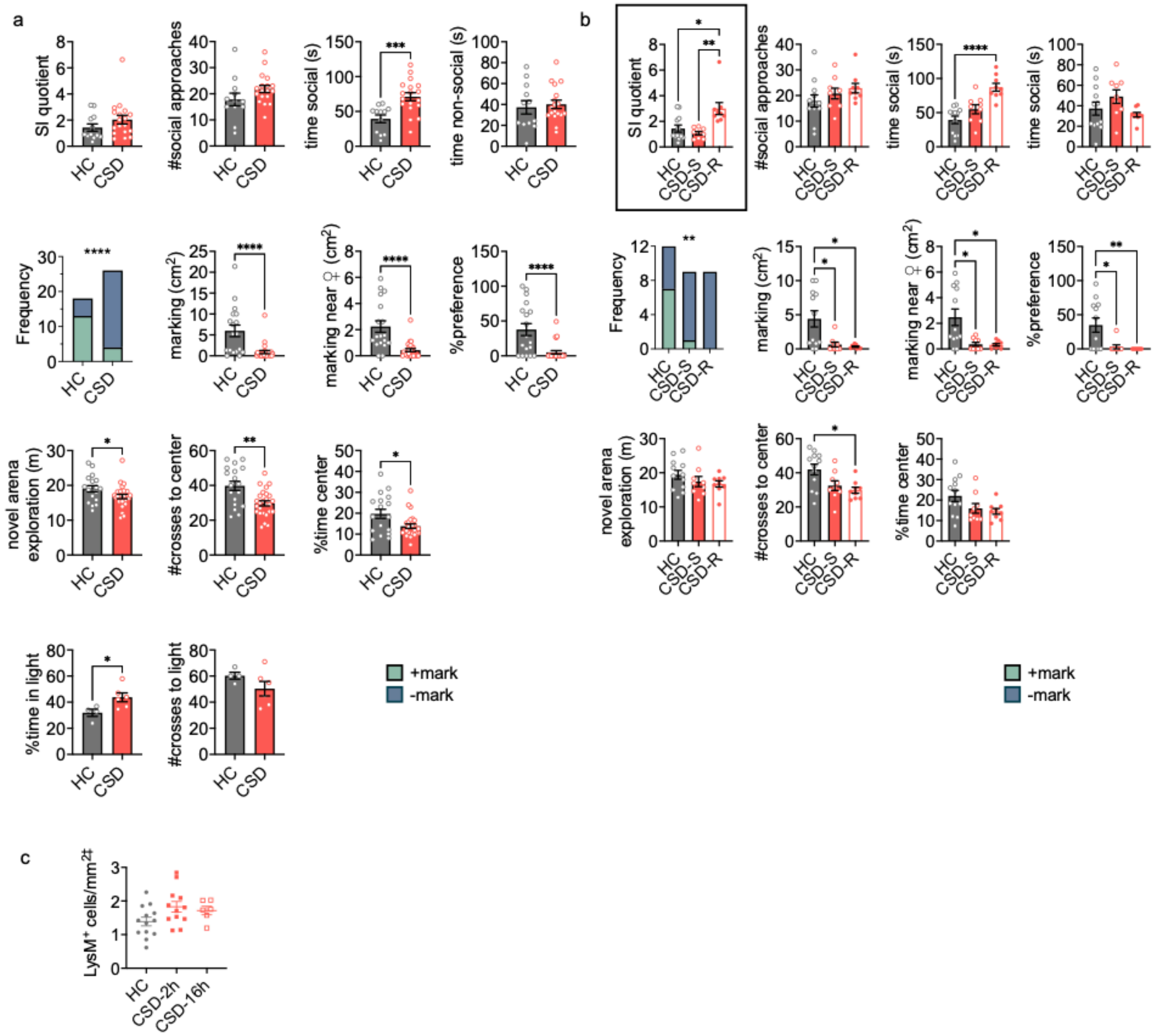

Figure S8: LysM$^{gfp/+}$ mice recapitulate expected behavioral phenotype following CSD stress. Note these animals were also used to generate data for **Figures 4C-F**, **S9**, and **S10A-D**. Behavioral testing began after 10 days of defeat stress; results are shown with all CSD animals combined (**a**), or with CSD animals stratified into susceptible vs resilient based on their performance in the social interaction (SI) test (**b**). By our non-formalized metrics, LysM mice were 'defeated' normally, and exhibited other signs typical of 'depressed' mice: e.g., poor coat quality. However, the social interaction test gave opposite to expected results. *Top row:* CSD mice spent more time in proximity to the social stimulus overall [(**a**) Mann Whitney test: ***p=0.0003, U=27. n_{HC}=12, n_{CSD}=18], though this seems to be driven mostly by CSD-R mice—which represented 50% of the CSD group [(**b**) Kruskal-Wallis test: ***P=0.0002, H=17.3; Dunn's post-hoc: ****$p_{HCvCSD\text{-}R}$<0.0001, $Z_{HCvCSD\text{-}R}$=4.2. n_{HC}=12, $n_{CSD\text{-}S}$=9, $n_{CSD\text{-}R}$=9]. Splitting mice into susceptible vs resilient based on SI quotient scores also did not generate the expected difference between HC and CSD-S [(**b**) Kruskal-Wallis test: **P=0.0023, H=12.2; Dunn's post-hoc: *$p_{HCvCSD\text{-}R}$=0.016, $Z_{HCvCSD\text{-}R}$=2.8; **$p_{CSD\text{-}SvCSD\text{-}R}$=0.0033, $Z_{CSD\text{-}SvCSD\text{-}R}$=3.3], but the HC group appeared to be less social than expected (SI scores < 2). This may be influenced by group housing of LysM$^{gfp/+}$ mice with their littermates post-weaning, as opposed to C57BL/6J mice that were purchased and pair-housed in cages with dividers upon arrival to our facility, though at this

time we cannot explain their behavior further. *Second row:* LysM$^{gfp/+}$ mice showed an expected 'anhedonic' response to CSD stress in the USM test: marking [(**a**) Fisher's exact test, ***p=0.0003. n_{HC}=18, n_{CSD}=26. (**b**) Fisher's exact test, **p=0.0057. n_{HC}=12, $n_{CSD\text{-}S}$=9, $n_{CSD\text{-}R}$=9], total urine area [(**a**) Mann Whitney test: ****p<0.001, U=76. n_{HC}=18, n_{CSD}=26. (**b**) Kruskal-Wallis test: *P=0.012, H=8.9; Dunn's post-hoc: *$p_{HCvCSD\text{-}S}$=0.031, $Z_{HCvCSD\text{-}S}$=2.6; *$p_{HCvCSD\text{-}R}$=0.040, $Z_{HCvCSD\text{-}R}$=2.5. n_{HC}=12, $n_{CSD\text{-}S}$=9, $n_{CSD\text{-}R}$=9], urine area near female scent [(**a**) Mann Whitney test: ****p<0.0001, U=64. n_{HC}=18, n_{CSD}=26. (**b**) Kruskal-Wallis test: **P=0.0074, H=9.8; Dunn's post-hoc: *$p_{HCvCSD\text{-}S}$=0.023, $Z_{HCvCSD\text{-}S}$=2.7; *$p_{HCvCSD\text{-}R}$=0.025, $Z_{HCvCSD\text{-}R}$=2.6. n_{HC}=12, $n_{CSD\text{-}S}$=9, $n_{CSD\text{-}R}$=9], and preference for female [(**a**) Mann Whitney test: ****p<0.0001, U=93. n_{HC}=18, n_{CSD}=26. (**b**) Kruskal-Wallis test: **P=0.0049, H=10.7; Dunn's post-hoc: *$p_{HCvCSD\text{-}S}$=0.037, $Z_{HCvCSD\text{-}S}$=2.5; **$p_{HCvCSD\text{-}R}$=0.0091, $Z_{HCvCSD\text{-}R}$=3.0. n_{HC}=12, $n_{CSD\text{-}S}$=9, $n_{CSD\text{-}R}$=9]. *Third row:* LysM$^{gfp/+}$ mice showed an expected anxiety-like behavioral response to CSD in the OF test: novel arena exploration [(**a**) Mann Whitney test: *p=0.045, U=150. n_{HC}=18, n_{CSD}=26], crosses to center [(**a**) Mann Whitney test: **p=0.0041, U=116. n_{HC}=18, n_{CSD}=26. (**b**) Kruskal-Wallis test: *P=0.028, H=7.2; Dunn's post-hoc: *$p_{HCvCSD\text{-}R}$=0.030, $Z_{HCvCSD\text{-}R}$=2.6. n_{HC}=12, $n_{CSD\text{-}S}$=9, $n_{CSD\text{-}R}$=9], and time spent in the center of the arena [(**a**) Mann Whitney test: *p=0.045, U=150. n_{HC}=18, n_{CSD}=26. (**b**) Kruskal-Wallis test: P=0.071, H=5.3. n_{HC}=12, $n_{CSD\text{-}S}$=9, $n_{CSD\text{-}R}$=9]. *Bottom row*: We did not test both L/D and SI in the same animals, so we cannot examine the effect of stress resiliency on this task. However, LysM$^{gfp/+}$ behavior was also in an unexpected direction on this test. **a**) CSD mice spent significantly more time in the light (Mann Whitney test: *p=0.038, U=2. n_{HC}=4, n_{CSD}=6). This may represent freezing, as crosses to light seemed to be in the expected direction (fewer in CSD). **c**) Quantification of total meningeal LysM-GFP^{+} cells 16 h post-CSD stress is consistent with the pattern seen in **Figure 3B** for C57BL/6J mice (One-way ANOVA: P=0.080, $F_{(2,28)}$=2.8; Tukey's post-hoc: $p_{CSD\text{-}2h/HC}$=0.073, $q_{CSD\text{-}2h/HC}$=3.2). ‡natural log-transformed. Data points represent individual mice. Univariate tests were two-tailed. Data shown as mean±SEM. Source data are provided as a Supplemental Source Data file.

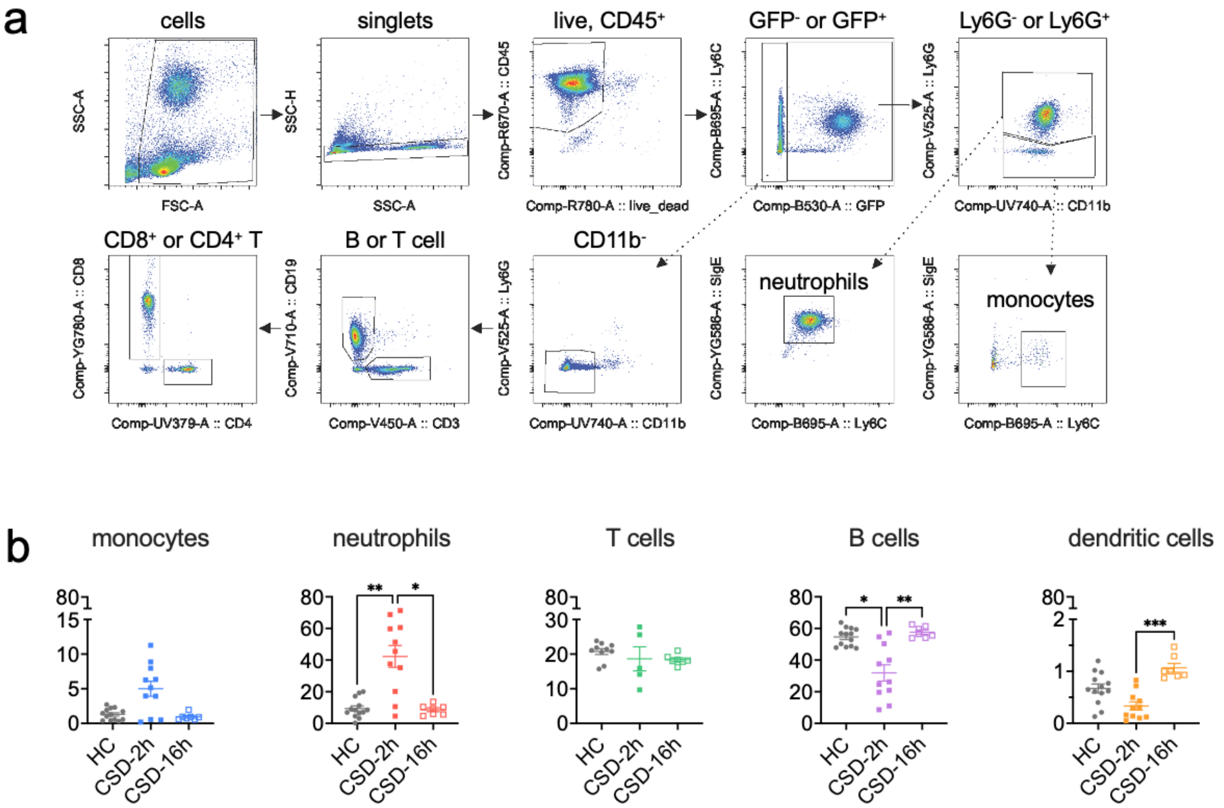

Figure S9: LysM$^{gfp/+}$ mice recapitulate blood immune responses to CSD stress seen in C57BL/6J mice. Note these animals were also used to generate data for **Figures 4C-F**, **S8**, and **S10A-D**. Compare results with **Figures 3B** and **S7B**. **a**) Gating strategy used to identify distinct white blood cell (WBC) populations collected from submandibular venous blood prior to tomato lectin (TomL) injection, exsanguination, and cardiac perfusion with formaldehyde. Value shown as a percent of live, CD45$^+$ cells. **b**) CSD increases blood monocyte (GFP^{int};CD11b$^+$;Ly6G$^-$;Ly6C^{hi}: Kruskal-Wallis test, P = 0.053, H = 5.9) and neutrophil (GFP^{hi};CD11b$^+$;Ly6G$^+$;Ly6C^{int}; Kruskal-Wallis test, **P = 0.0016, H = 12.9) levels. Blood neutrophil levels return to control levels within 16h of CSD stress cessation (Dunn's post-hoc: **$p_{HCvCSD\text{-}2h}$ = 0.0029, $Z_{HCvCSD\text{-}2h}$ = 3.3; *$p_{CSD\text{-}2h\ v\ CSD\text{-}16h}$ = 0.017, $Z_{CSD\text{-}2h\ v\ CSD\text{-}16h}$ = 2.8). CSD stress led to a decreased percentage of B cells that recovered within 16h post-CSD (defined as GFP$^-$;CD11b$^-$;CD19$^+$: Kruskal-Wallis test, **P = 0.0018, H = 12.7; Dunn's post-hoc: *$p_{HCvCSD\text{-}2h}$ = 0.013, $Z_{HCvCSD\text{-}2h}$ = 2.9; **$p_{CSD\text{-}2h\ v\ CSD\text{-}16h}$ = 0.0041, $Z_{CSD\text{-}2h\ v\ CSD\text{-}16h}$ = 3.2). With 16 h of recovery from CSD, dendritic cell levels were elevated relatively to the CSD+2 h timepoint (CD11c$^+$;MHCII$^+$;GFP-mixed: Kruskal-Wallis test, ***P = 0.0003, H = 16.0; Dunn's post-hoc: ***$p_{CSD\text{-}2h\ v\ CSD\text{-}16h}$ = 0.0002, $Z_{CSD\text{-}2h\ v\ CSD\text{-}16h}$ = 4.0). n_{HC} = 13, $n_{CSD\text{-}2h}$ = 11, $n_{CSD\text{-}16h}$ = 7. Data points in (**b**) represent individual mice. Data shown as mean ± SEM. Source data are provided as a Supplemental Source Data file.

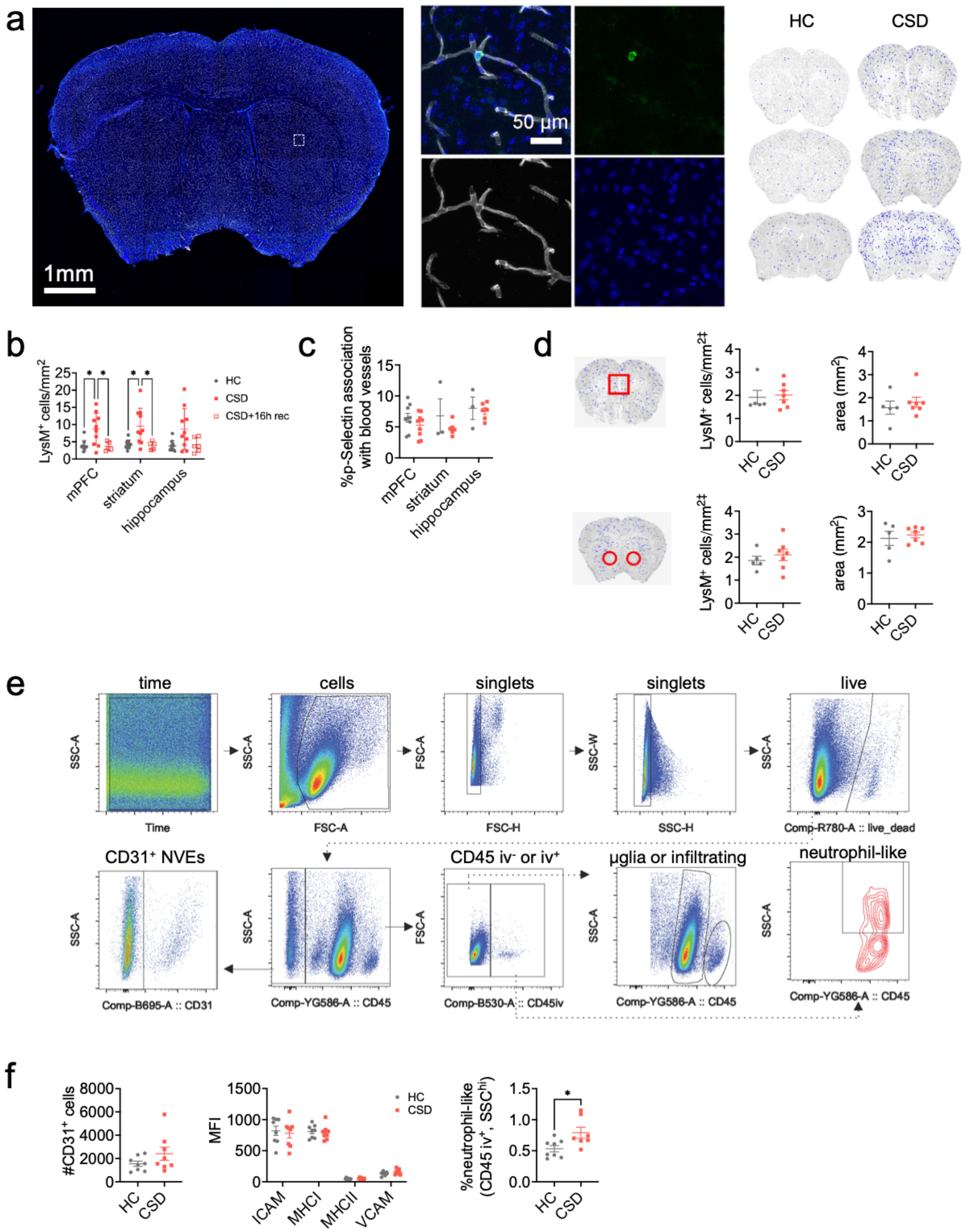

Figure S10: CSD increases LysM$^+$ myeloid cells in neurovasculature, but not brain parenchyma. **a**) Confocal images of GFP$^+$ cells in brains from LysM$^{gfp/+}$ mice (note these are the same mice presented in **Figures 4C-F** and **S8-9**). Representative coronal section containing striatum showing nuclei (DAPI, blue), blood vessels (TomL, white), and LysM$^+$ cells (GFP,

green). *Left:* 20x magnification composite with online stitching. *Middle (clockwise from top left)*: 60x oil magnification, three color merge showing $LysM^+$ cells contained within blood vessels, GFP signal alone, nuclei alone, and blood vessels alone. *Right*: Representative, inverted images of sections containing medial prefrontal cortex (mPFC, *top*), striatum (*middle*), or hippocampus (*bottom*). Images show blood vessels (gray) overlaid with hand-counted $LysM^+$ cells (blue dots). **b**) Quantification of $LysM^+$ cells per mm^2 of tissue analyzed show a main effect of CSD stress on greater $LysM^+$ cell accumulation in blood vessels. Within 16 h of stress cessation, LysM-GFP^+ cell levels had returned to control levels (REML: main effect of group, $**p$=0.0042, $F_{(2,23)}$=7.0. Šídák's post hoc. *mPFC:* $*p_{HCvCSD\text{-}2h}$=0.024, $q_{HCvCSD\text{-}2h}$=4.4; $*p_{CSD\text{-}2h/CSD\text{-}16h}$=0.021, $q_{CSD\text{-}2h/CSD\text{-}16h}$=4.5. *Striatum*: $*p_{HCvCSD\text{-}2h}$=0.025, $q_{HCvCSD\text{-}2h}$=4.5; $*p_{CSD\text{-}2h/CSD\text{-}16h}$=0.019, $q_{CSD\text{-}2h/CSD\text{-}16h}$=4.6). n_{HC}=10, $n_{CSD\text{-}2h}$=10, $n_{CSD\text{-}16h}$=5 mPFC/striatum or 6 hippocampus. No $LysM^+$ cells were observed outside of blood vessels, i.e. in brain parenchyma. **c**) We counter-stained a subset of samples with antibodies to p-Selectin, a protein that 'captures' circulating neutrophils onto vascular endothelial cells[2], but found no significant group differences in p-Selectin co-localization with blood vessels (n_{HC}=3 striatum/hippocampus or 10 mPFC, n_{CSD}=6 striatum/hippocampus or 9 mPFC). **d**) In a subset of samples, we looked at LysM-GFP+ myeloid cell sticking in the prelimbic cortex (PrL) and nucleus accumbens (NAc) given previous work highlighting a key role for these areas in stress-related anhedonic behavioral changes[3–5]. *Left*: representative section showing area of tissue examined for neutrophil counting. *Middle*: No differences between HC and CSD mice in LysM-GFP+ cell vascular sticking in either region of interest (ROI). GFP+ cell counts were normalized to the ROI investigated. ‡natural log-transformed values to improve normality. *Right:* no differences in the ROI areas examined between groups. n_{HC}=5, n_{CSD}=7. **e**) Neurovascular endothelial (NVE) isolations were performed to examine expression of other adhesion or otherwise anchoring molecules in C57BL/6J wild type mice. Samples were stained for flow cytometric analysis and gated according to this strategy. **f**) *Left*: Comparable yields of $CD31^+$;$CD45^-$ NVEs were obtained (n_{HC}=8, n_{CSD}=8). *Middle*: NVEs were examined for expression of the indicated adhesion or antigen-presenting molecules; no differences in expression of these proteins were observed between groups. *Right*: 'Neutrophil-like' (i.e., SSC^{hi};$CD45^{hi}$) cells were pulled down in our NVE isolations more frequently for CSD mice than HC mice, consistent with our observations in fixed tissue. Data points in (**b,c,d,f**) represent individual mice. Univariate tests were two-tailed. HC = home cage, CSD = chronic social defeat stress. Data shown as mean±SEM. Source data are provided as a Supplemental Source Data file.

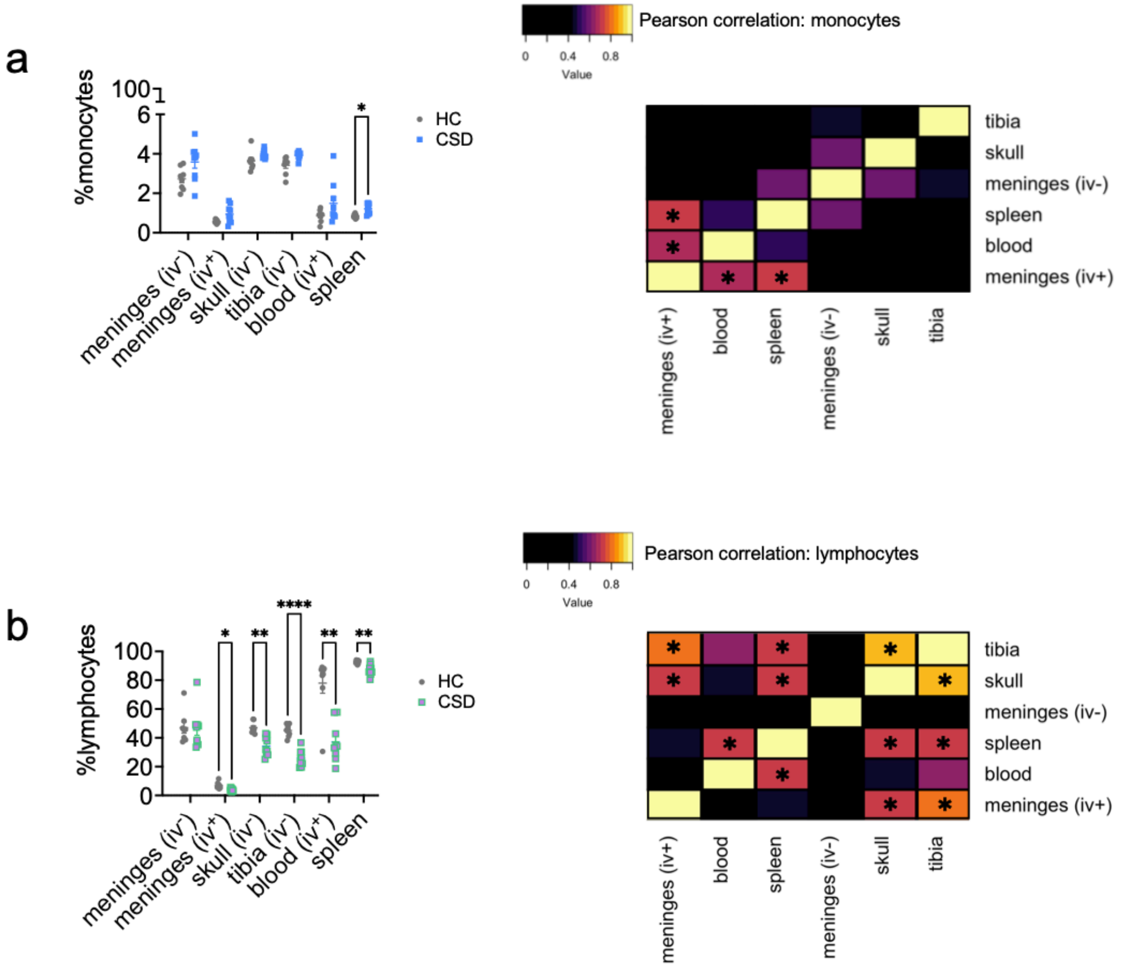

***Figure S11**:* Cross-tissue effects of CSD on monocytes and lymphocytes. Unlike for neutrophils (**Figure 5A**), meningeal monocyte and lymphocyte populations do not correlate with their corresponding populations in skull bone marrow in C57BL/6J mice. **a**) Flow cytometry shows a main effect of stress on monocyte levels throughout the body, though only the spleen was significant with multiple comparisons corrections (2-way ANOVA with RM measures, main effect of group, ***p = 0.0002, $F_{(1,15)}$ = 24.7. Šídák's post hoc: *spleen* iv^+, *p = 0.012, t = 4.1). Supervised hierarchical clustering, based on neutrophil clustering pattern, shows iv^+ meningeal, blood, and spleen monocytes significantly correlate with each other, but iv^- meningeal and bone marrow monocyte levels do not. This is in contrast to the pattern seen in neutrophils, shown in **Figure 5B**. **b**) To examine inter-tissue relationships between lymphocytes in this data set, which did not include CD3 or CD19 markers, we gated on $CD11b^-$;FSC^{lo}; SSC^{lo} cells. As with other studies, we saw decreased lymphocytes (likely representing B cells) across multiple tissues (2-way ANOVA with RM measures, main effect of group, ****p < 0.0001, $F_{(1,15)}$ = 85.9. Šídák's post hoc: *meninges* iv^+, *p= 0.038, t = 3.5; *skull* iv^-, **p= 0.0016, t = 4.9; *tibia* iv^-, ****p < 0.0001, t = 8.0; *blood* iv^+, **p< 0.0020, t = 4.9; *spleen* iv^+, **p< 0.0077, t = 4.5). Supervised hierarchical clustering, based on neutrophil clustering pattern, shows iv^+ meningeal relationships, but no iv^- meningeal relationships, in contrast to **Figure 5B**. n_{HC} = 8, n_{CSD} = 9; for blood, n_{HC} = 7. Data points represent individual mice. Data shown as mean ± SEM. Source data are provided as a Supplemental Source Data file.

a Marker genes by % of cells expressing gene

Microglia	Preneutrophils	Neutrophils	Monocytes Ly6Chi	Monocytes NOS	Border-associated macrophage	Perivascular macrophage (Cd206)	cDCs	pDCs	Mast cells and others	Endothelial and others	Fibroblasts and other	Olfactory neurons	Pro B cells and other precursors	Pre B cells	Mature B cells 1	Mature B cells 2	T cells 1	T cells 2	NK cells	Erythrocytes
P2ry12	Gm10282	Cxcr2	Asf1b	Fn1	Ms4a7	Mrc1	Cd209a	Ccr9	Cdk6	Crip2	Col1a2	Elavl3	Myl4	Arl5c	Ms4a1	Fcmr	Cd3g	Cd3g	Klrb1c	Trim10
Gpr34	1700020L24Rik	Hdc	Cdca3	Ms4a8a	Cxcl16	Dab2	Tnip3	Cox6a2	Cpa3	Krt18	Rspo3	Fstl5	Mzb1	Fcrla	Tnfrsf13c	Gm31243	Il7r	Cd3d	Klre1	Gypa
Siglech	Orm1	Trem3	Ccna2	Clec4a1	Slamf9	Stab1	Clec4b1	Sh3bgr	Cst7	Flt1	Serpinh1	Gnal	Gm30211	Ebf1	Siglecg	H2-DMb2	Thy1	Thy1	Klrk1	Slc4a1
Tmem119	Ms4a3	Slc7a11	Racgap1	Clec4a3	C3ar1	Ms4a7	H2-DMb1	Cd300c	Gata2	Igfbp7	Aebp1	Scn9a	Ezh2	Tifa	Fcmr	Ms4a1	Lat	Ms4a4b	Klrd1	Alas2
Olfml3	Spc25	Mmp9	Mki67	Ccr2	H2-DMb1	Cbr2	Cbfa2t3	Siglech	Ms4a2	Timp3	Cald1	Sult1d1	Bcl7a	Vpreb3	Cd72	Bank1	Cd3d	Lck	Xcl1	Tspo2
Fcrls	Ccnb2	Fpr2	Smc2	Ms4a4c	Ctsc	Pf4	Slamf7	Klk1	Csrp3	Hspb1	Pcolce	Clgn	Smarca4	Pafah1b3	Spib	Cd79a	Skap1	Gimap4	Ncr1	Cldn13
Crybb1	Cdkn3	Pilra	Knstrn	F13a1	Tmem176a	Maf	Ccr2	Cd7	Srm	Ly6c1	Efemp1	Snap25	Sox4	Cd72	Cd79a	Ebf1	Cxcr6	Skap1	Txk	Rhd
Mafb	Inhba	Mxd1	Tacc3	AI839979	Lgmn	Folr2	Ccnd1	Upb1	Sfxn1	Tcf4	Plpp3	Flrt1	Lockd	Siglecg	Ebf1	H2-Ob	Bcl11b	Cd8b1	Ctsw	Fech
Hpgds	Cdca8	Il1r2	Ccnb2	Mcub	Tmem176b	Gas6	Mgl2	Runx2	Rcl1	Krt8	Igfbp5	Ttll7	Igll1	Sox4	Vpreb3	Cd79b	H2-Q7	Gimap3	Cd7	Sox6
Ecscr	Mki67	Chil1	Spc24	Cd300a	Aif1	Lyve1	Pid1	P2ry14	Gnl3	Fxyd6	Itm2a	Pcolce2	Akap12	Cnp	Pax5	Gm8369	Ccnd2	Ctsw	Ms4a4b	Snca

b Marker genes by level of expression

Microglia	Preneutrophils	Neutrophils	Monocytes Ly6Chi	Monocytes NOS	Border-associated macrophage	Perivascular macrophage (Cd206)	cDCs	pDCs	Mast cells and others	Endothelial and others	Fibroblasts and other	Olfactory neurons	Pro B cells and other precursors	Pre B cells	Mature B cells 1	Mature B cells 2	T cells 1	T cells 2	NK cells	Erythrocytes
Cst3	Camp	S100a8	Pclaf	Lyz2	Cd74	Apoe	H2-DMa	Irf8	Rpl15	Crip2	Malat1	Calm1	Ptma	Vpreb3	Ly6d	Rpl18a	Rpl17	Cd3d	Nkg7	Hba-a1
Hexb	Trem3	Retnlg	Lgals1	S100a4	H2-Aa	C1qa	H2-DMb1	Rpl31	Rps12	Ly6a	Mgp	Omp	Ptprcap	Ebf1	Cd79a	Rps19	Rps14	Rps15a	AW112010	Hbb-bs
Lgmn	H2afz	Cxcr2	S100a10	Lgals3	H2-Eb1	Pf4	Rps11	Plac8	Cmtm7	Krt18	Plpp3	Gng13	Stmn1	Chchd10	Cd79b	Fcmr	Tpt1	Hcst	Ccl5	Hbb-bt
P2ry12	Ngp	Bmx	Ly6c2	Ifitm3	H2-Ab1	Selenop	Plbd1	Bst2	Rpl14	Hspb1	Serpinf1	Nsg1	Tubb5	Arl5c	Ms4a1	Rps27	Rplp1	Cd3g	Klrk1	Car2
Tmem119	Hmgb2	S100a9	Tmsb10	F13a1	Tmem176b	Ctsb	Gm2a	Rpl10	Srgn	Ly6c1	Id3	Stoml3	Hmgb1	Dnajc7	Siglecg	Ltb	Il7r	Rps16	Klrb1c	Gpx1
C1qc	Hmgn2	Cxcl2	Tuba1b	Ms4a6c	Aif1	Dab2	Rps9	Siglech	Ifitm1	Tm4sf1	Igfbp5	Map1b	H2afv	Tifa	Btg1	Rpl13	Rpl36a	B2m	Ncr1	Hba-a2
Ctss	Wfdc21	Gsr	Pycard	Napsa	Ctsh	Cd68	Tnip3	Cox6a2	Ctsg	Cmtm8	1500015O10Rik	Tuba1a	Mzb1	Xrcc6	Ifi30	H2-DMb2	Rpl19	Gimap4	Klrd1	Prdx2
Sparc	Pglyrp1	Slc7a11	Ppia	Fn1	Tmem176a	Maf	Naaa	Sec61b	Npm1	Flt1	Col1a2	Calm2	Smarca4	Pafah1b3	Cd37	Rps29	Rplp0	Rpl13a	Xcl1	Gypa
Selplg	Lcn2	Itgam	Ran	Psap	Slamf9	Cbr2	Cdkn1a	Rpl36al	Ier3	Arhgap31	Rbp1	Tshz2	Pgls	Rhoh	Tnfrsf13c	Gm31243	Rps5	Ms4a4b	Gzma	Cd24a
C1qb	Anxa1	B430306N03Rik	Crip1	Smpdl3a	Fth1	Ftl1	Cd209a	Tcf4	Cst7	Sparcl1	Klf9	Plekhb1	Pkig	Fam53b	Cd72	Bank1	Cd163l1	Rps13	Id2	Alas2

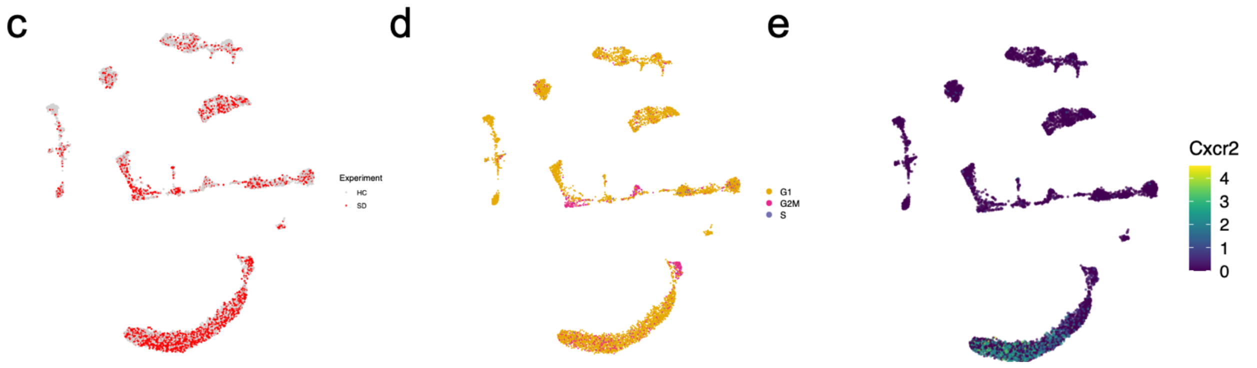

Figure 12: Cell-type specific gene expression analysis via the 10x Genomics platform was used to identify unique cell populations in the meninges. Data shown here indicate cell type-specific genes ranked for either **a**) genes for which frequency of expression in the cluster is most predictive of cluster (TF-IDF method) or **b**) differential expression between clusters (see **Methods**). **c**) Home cage (HC) control and chronic social defeat (CSD) stress samples were barcoded then pooled for analysis and clustering shown in **Figure 6C**. Here, group status for individual cells is shown in a uniform manifold approximation and projection (UMAP), with HC in gray and CSD in red. **d**) Meningeal single cell UMAP, colored by inferred cell cycle phase (G1, beginning of interphase; S, synthesis phase; or G2M, end of interphase or mitosis, see **Methods**). **e**) *Cxcr2* expression, which is highly specific to neutrophils[6], overlain on the meningeal UMAP. As *Cxcr2* expression was differentially expressed, we explored this as a potential signaling mechanism further; see **Figures S10C-E**. Data points represent individual cells from pooled sample.

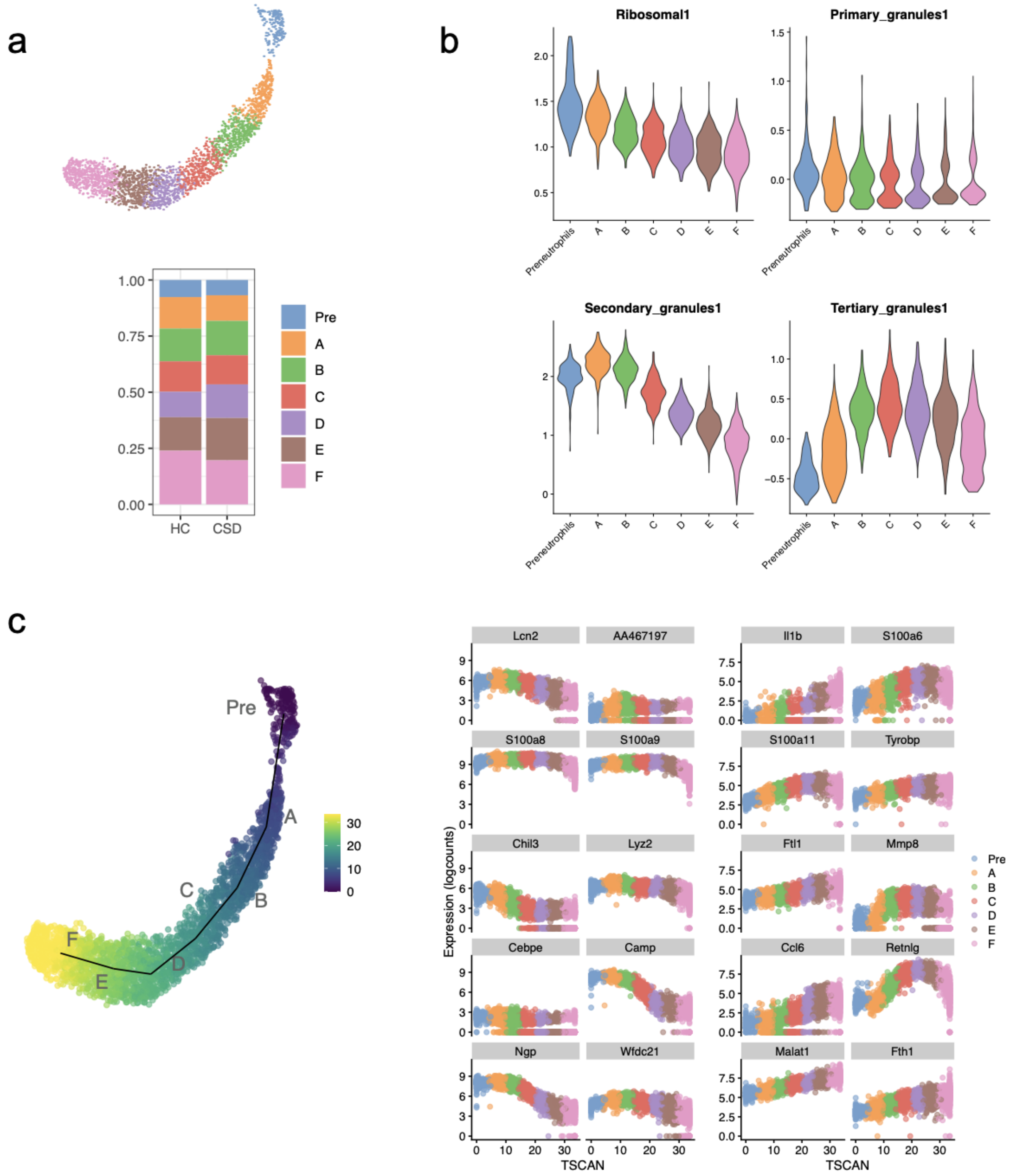

Figure S13: Neutrophil population heterogeneity represents various states of maturation. **a**) Reclustering neutrophils alone generated 6 subclusters (plotted here with ‘preneutrophils’). Relative proportions of each of these subclusters were similar between groups. **b**) Expression of gene sets corresponding to primary, secondary and tertiary granules within each neutrophil cluster, corresponding to neutrophil maturation (A to F); gene lists from[7]. **c**) *Left:* Pseudotime analysis (see **Methods**) of the meningeal cluster suggests that the subclusters represent a gradient of neutrophil maturation (less mature to more mature, moving away from preneutrophils). *Right:* Genes most significantly down-regulated (left hand side) and up-

regulated (right hand side) with pseudotime gradient, colored by cluster. Based on relatively high expression of *Chil3, Camp,* and *Ngp*, as well as their proximity to an adjacent 'preneutrophil' cluster, subclusters A-C most likely represent immature, bone-marrow like neutrophils that may have either recently migrated from the skull via diploic veins connected to meninges[8–11] or that may be skull bone marrow resident cells and thus a technical artifact from meningeal tissue preparation. Conversely, increased expression of *Ccl6* and *Il1b* in subclusters D-F is consistent with the transcriptome of mature blood and tissue neutrophils[7,12,13]. Data points represent individual cells from pooled sample. HC = home cage, CSD = chronic social defeat stress.

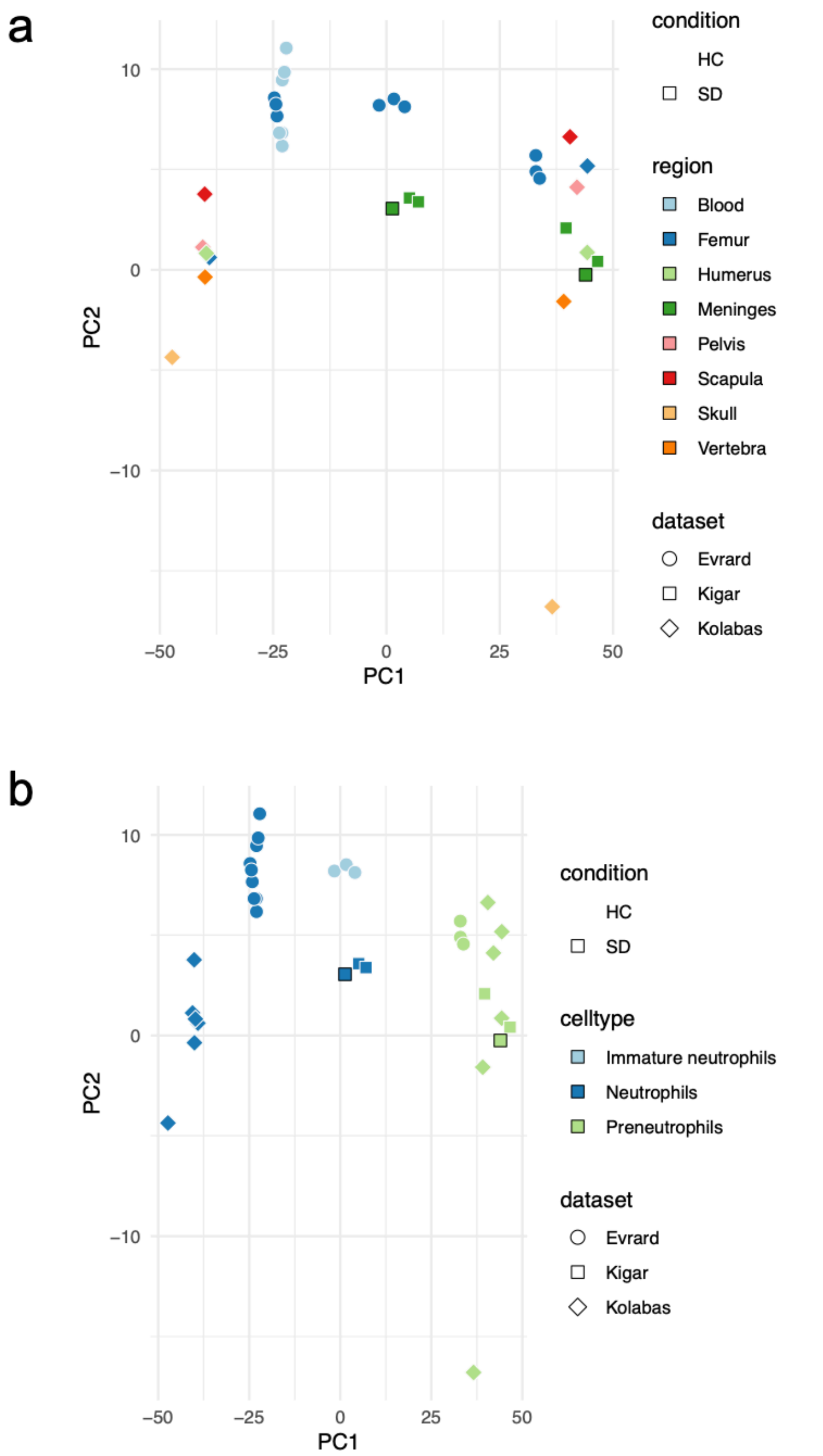

Figure S14: Principal Component Analysis (PCA) plots visualize the transcriptomic similarity of neutrophil and preneutrophil populations across tissues (blood, meninges and various bone marrow locations), cell types (neutrophils and preneutrophils), condition (HC, control and CSD, stress), and dataset. We integrated our meningeal dataset with two publicly available mouse

RNA-seq datasets[7,10] containing neutrophils and pre-neutrophils from blood and multiple bone marrow sites. Variance stabilising transform was applied to the bulk (Evrard) and pseudobulked (our meningeal data and Kolabas) samples (see **Methods**). PCA was performed on Kolabas pseudobulk samples using the 20% most variable genes intersected with genes present in all three datasets. VST matrices from our data and Evrard's were quantile-normalized and projected into this PCA space. **a**) PCA plot highlighting tissue origin (predominantly PC2) **b**) PCA plot highlighting cell types (reflected by PC1). Meningeal pre-neutrophils cluster with pre-neutrophils from Kolabas and Evrard samples but meningeal neutrophils are most similar to bone marrow immature neutrophils. HC = control (white outlines), SD = social defeat (black outlines). Data points represent individual samples.

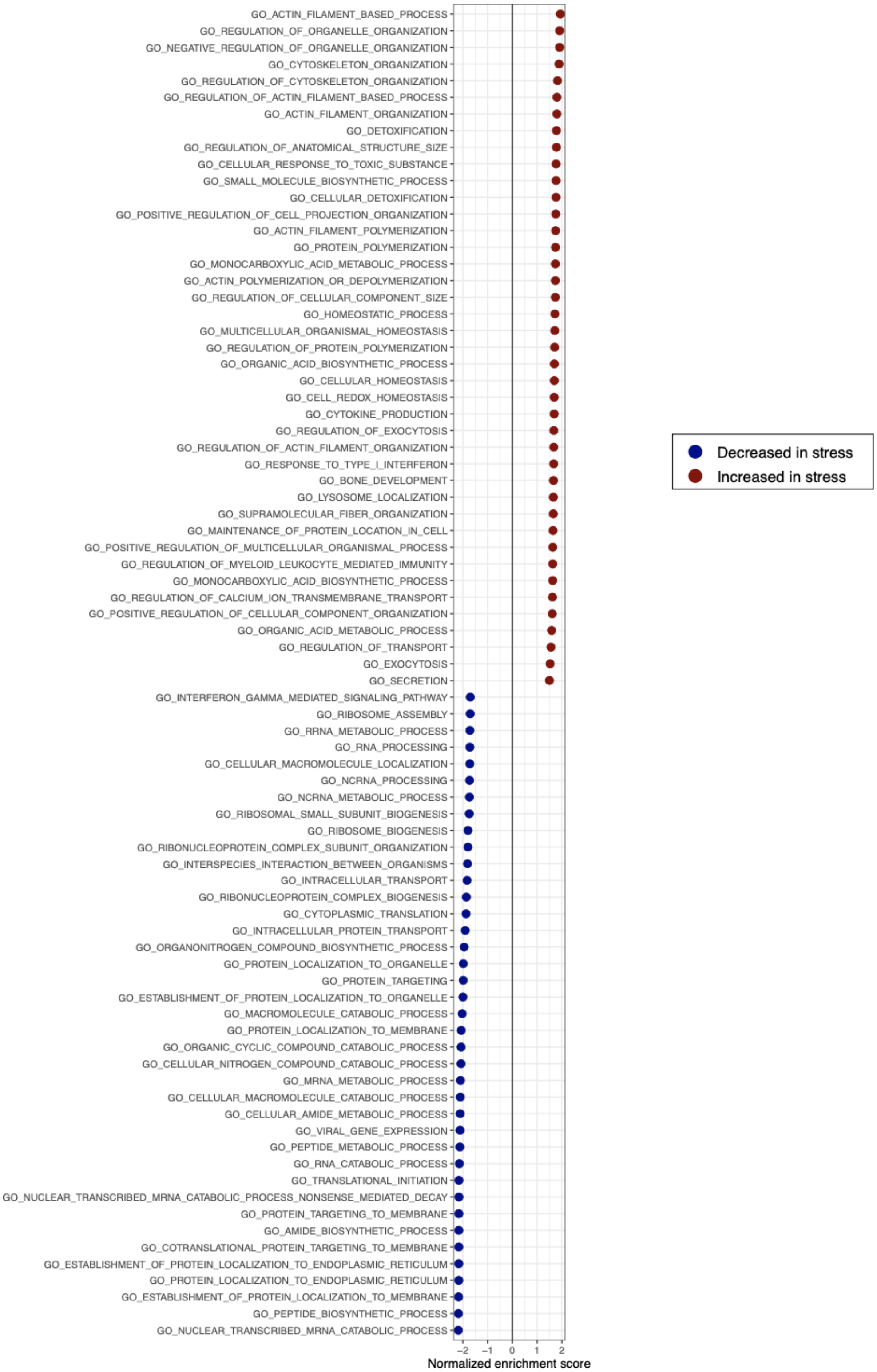

Figure S15: Identification of biological pathways associated with differentially expressed genes in chronic social defeat stressed vs control animals via gene set enrichment analysis (GSEA). Plot shows gene ontology (GO) Biological Process pathways significant at FDR < 0.05. We systematically explored several of the top pathways using biological assays, including those

related to actin and cell size (**Figure 6G**, **Figure S16**), cytokine production **(Figure S17**), and reactive oxygen species (ROS) production (**Figure S18**).

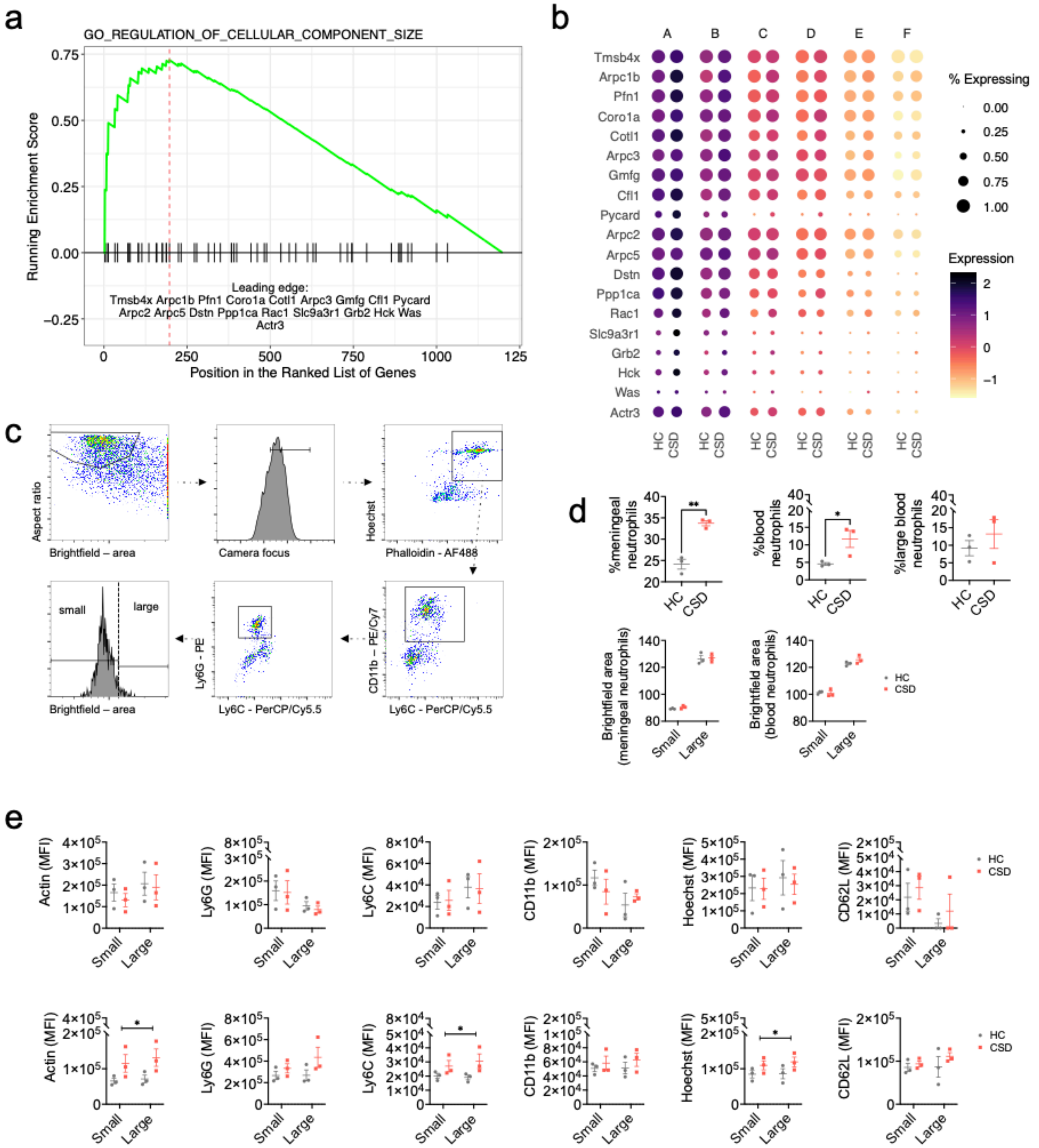

Figure S16: Actin-based gene signature in meningeal and blood neutrophils. **a**) Escarpment plot showing enrichment of GO: Regulation of cellular component size pathway in meningeal neutrophils; leading edge genes are listed as overlay. **b**) Dot plot showing expression of leading-edge genes in each neutrophil subcluster for all genes comprising the GO: Regulation of cellular component size pathway. Expression is scaled to mean±standard deviation. Leading edge genes included 5 of 7 subunits comprising the Arp2/3 complex (*Arpc1b, Arpc2, Arpc3, Arpc5, Actr3*) and the small GTPase *Rac1,* which works together with Arp2/3 in neutrophils to promote actin filament branching and cell migration in response to danger- and pathogen-associated molecular patterns (DAMPs/PAMPs)[14,15]. In neutrophil subclusters B, D, and E, CSD neutrophils expressed more *Tmsb4x,* which sequesters actin monomers, prevents rigid

filamentous actin polymerization, and facilitates cell motility[16]. There was a notable gradient in the expression of actin process-related transcripts across subclusters, with highest expression in the less mature neutrophils and lowest expression in the most mature neutrophils. This could reflect extravasation of maturing neutrophils as they migrate from proximal skull bone marrow to the meninges. **c**) Gating strategy for neutrophil identification in the Amnis ImageStream study; note that the "Brightfield" parameter permits microscopic analysis of cell area. Cells were stained with phalloidin to label actin filaments, Hoechst dye to label DNA, and with lineage markers to identify neutrophils. **d**) *Top*: Validation of increased meningeal (Unpaired t test: $^{**}p=0.0018$, $t=7.4$, $df=4$) and blood (Unpaired t test: $^{*}p=0.045$, $t=2.9$, $df=4$) neutrophils for animals in this study ($n_{HC}=3$, $n_{CSD}=3$). No difference in 'enlarged' blood neutrophil population (compare with **Figure 6G**). NB: tests were two-tailed. *Bottom*: Stratification of neutrophils by size revealed no further differences in area between groups. **e**) Median fluorescence intensity (MFI) for all parameters other than brightfield (see **d**) in the Amnis study. *Top:* meningeal neutrophil parameters. *Bottom:* blood neutrophil parameters. In meningeal neutrophils, there was a trend for lower Ly6G and CD62L (Ly6G: main effect of cell size, $p=0.06$, $F_{(1,9)}=4.5$; CD62L: main effect of cell size, $p=0.07$, $F_{(1,9)}=4.3$). In blood neutrophils, actin staining intensity was increased in both large and small subsets, suggesting increased rigidity (main effect of group, $^{*}p=0.013$, $F_{(1,9)}=9.6$. Tukey's post hoc, $q=4.4$, $p=0.052$). There was also an effect of CSD stress on Ly6C MFI (main effect of group, $^{*}p=0.024$, $F_{(1,9)}=7.4$. Tukey's post hoc, $q=3.9$, $p=0.091$) as well as increased MFI for the DNA-binding dye, Hoechst (main effect of group, $^{*}p=0.040$, $F_{(1,9)}=5.8$. Tukey's post hoc, $q=3.4$, $p=0.15$). Data points in (**d,e**) represent individual mice. HC=home cage, CSD=chronic social defeat stress. Data shown as mean±SEM. Source data are provided as a Supplemental Source Data file.

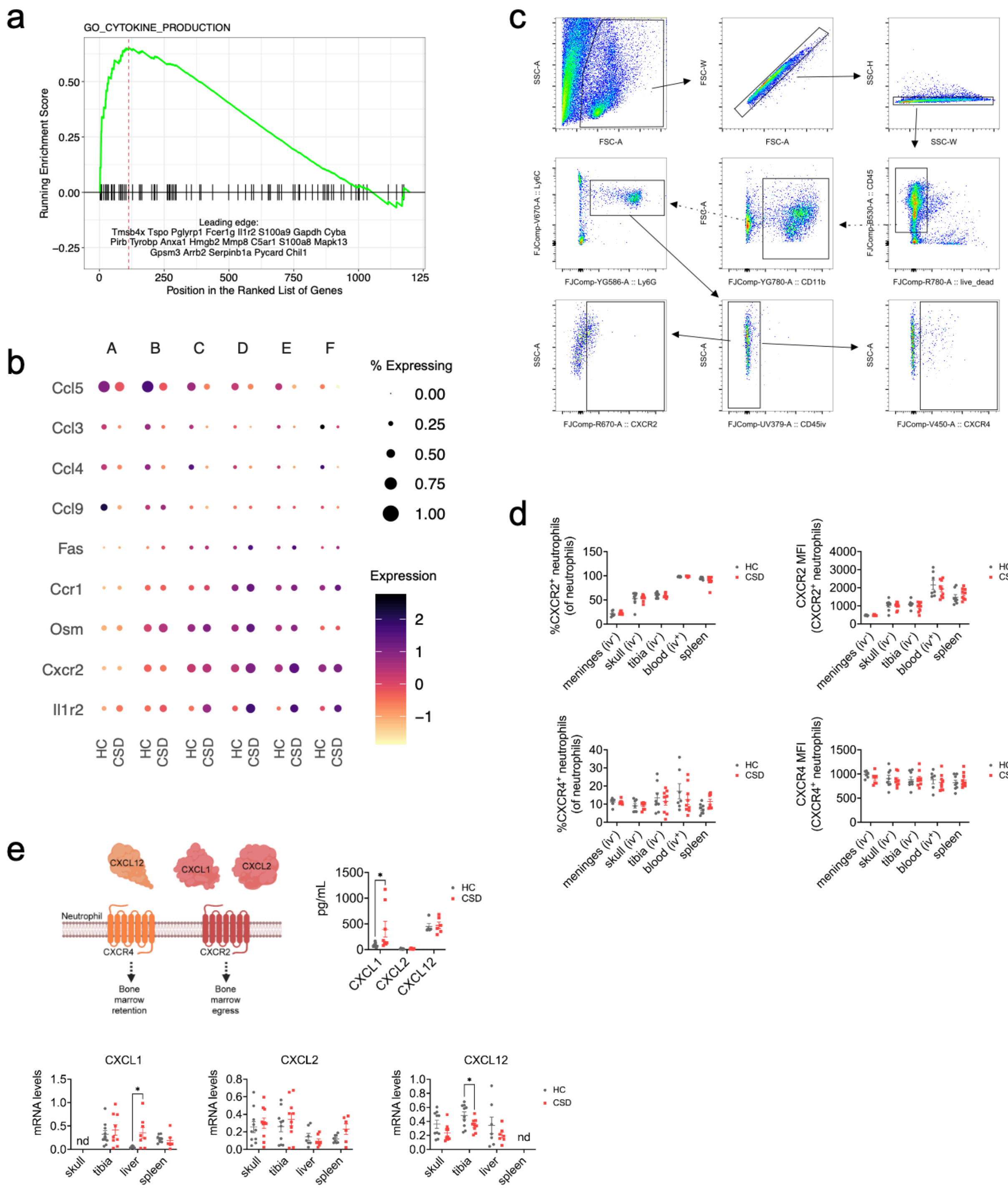

Figure S17: Cytokine & chemokine signaling in meningeal neutrophils and peripheral immune tissues. **a**) Escarpment plot showing enrichment of GO:Cytokine production pathway in meningeal neutrophils; leading edge genes are listed as overlay. **b**) Dot plot showing genes from KEGG pathway Cytokine-cytokine receptor interaction that were differentially expressed at FDR p<0.05 in the pooled neutrophil cluster; LFC for these genes is shown per neutrophil subcluster. *Pycard*–also known as ASC–is a protein involved in formation of the IL-1β-producing inflammasome[17], and *Il1r2*–the decoy receptor for IL-1β which becomes upregulated on neutrophils in situations of acute inflammation like sepsis[18,19]. There were also CSD-associated

changes in specific cytokines and cytokine receptors in neutrophils, including increased expression of *Fas*, *Ccr1*, and *Osm. Fas* is a death receptor whose expression on mature neutrophils makes them uniquely primed for rapid apoptosis[20]. *Ccr1* signaling in neutrophils promotes phagocytic activity in tissue-resident macrophages[21,22], which may facilitate clearance of Fas-mediated dying neutrophils. Finally, neutrophil expression of *Osm* leads to the recruitment of more neutrophils[23]; this feed-forward mechanism is seen in chronic inflammatory disorders like rheumatoid arthritis[24]. **c**) *Cxcr2* expression was also significantly increased in CSD neutrophils; because it regulates neutrophil egress from bone marrow under conditions of stress[25], we further investigated this pathway. Gating strategy for assessing CXCR2$^+$ and CXCR4$^+$ neutrophil staining; a fluorescence minus one (FMO) control was used to set the negative gates for each tissue on each day of data acquisition. **d**) No significant differences between HC and CSD seen in %CXCR-2,4$^+$ neutrophils in any tissues (normalized to live, CD45$^+$ neutrophils). n_{HC}=8 (in blood, n=7), n_{CSD}=9. Note: these data were also used to generate the correlation matrices and hierarchical clustering maps shown in **Figures 5** and **S11**. **e**) *Top*: Schematic showing typical chemokine regulation of neutrophil retention or egress from bone marrow to blood. Specifically, CXCL12 binding to CXCR4 maintains neutrophil localization to bone marrow. Both CXCL1 and CXCL2 binding to CXCR2 cause neutrophil egress out of the bone marrow. 3 independent ELISA assays were run; results showed increased circulating levels of CXCL1 in plasma (Mann Whitney test: **p=0.0079, U=9, n_{HC}=9, n_{CSD}=8). There were no effects of CSD on plasma CXCL2 (n_{HC}=7, n_{CSD}=6) or CXCL12 (n_{HC}=5, n_{CSD}=6). *Bottom*: qPCR expression of chemokines in immunoregulatory tissues (*Liver:* n_{HC}=7, n_{CSD}=8. *Skull*: n_{HC}=10, n_{CSD}=10. *Spleen*: n_{HC}=8, n_{CSD}=6. *Tibia*: n_{HC}=10, n_{CSD}=10). There was a significant increase in liver *Cxcl1* mRNA expression from CSD mice (Mann Whitney test: *p=0.039, U=8), and no detection in skull bone marrow (n_{HC}=10, n_{CSD}=10). No effects of CSD stress were observed in *Cxcl2* mRNA. CSD stress significantly decreased CXCL12 expression in tibia bone marrow (Unpaired t test: *p=0.037, t=2.3, df=17); there was also a trend towards less expression in skull bone marrow (Unpaired t test: p=0.078, t=1.9, df=18). No CXCL12 expression was detected in the spleen. Data points in (**d,e**) represent individual mice. Data shown as mean±SEM. HC=home cage, CSD=chronic social defeat stress, LFC=log fold change. Source data are provided as a Supplemental Source Data file. Schematic figure created in BioRender. Kigar, S. (2025) https://BioRender.com/tgefnj3

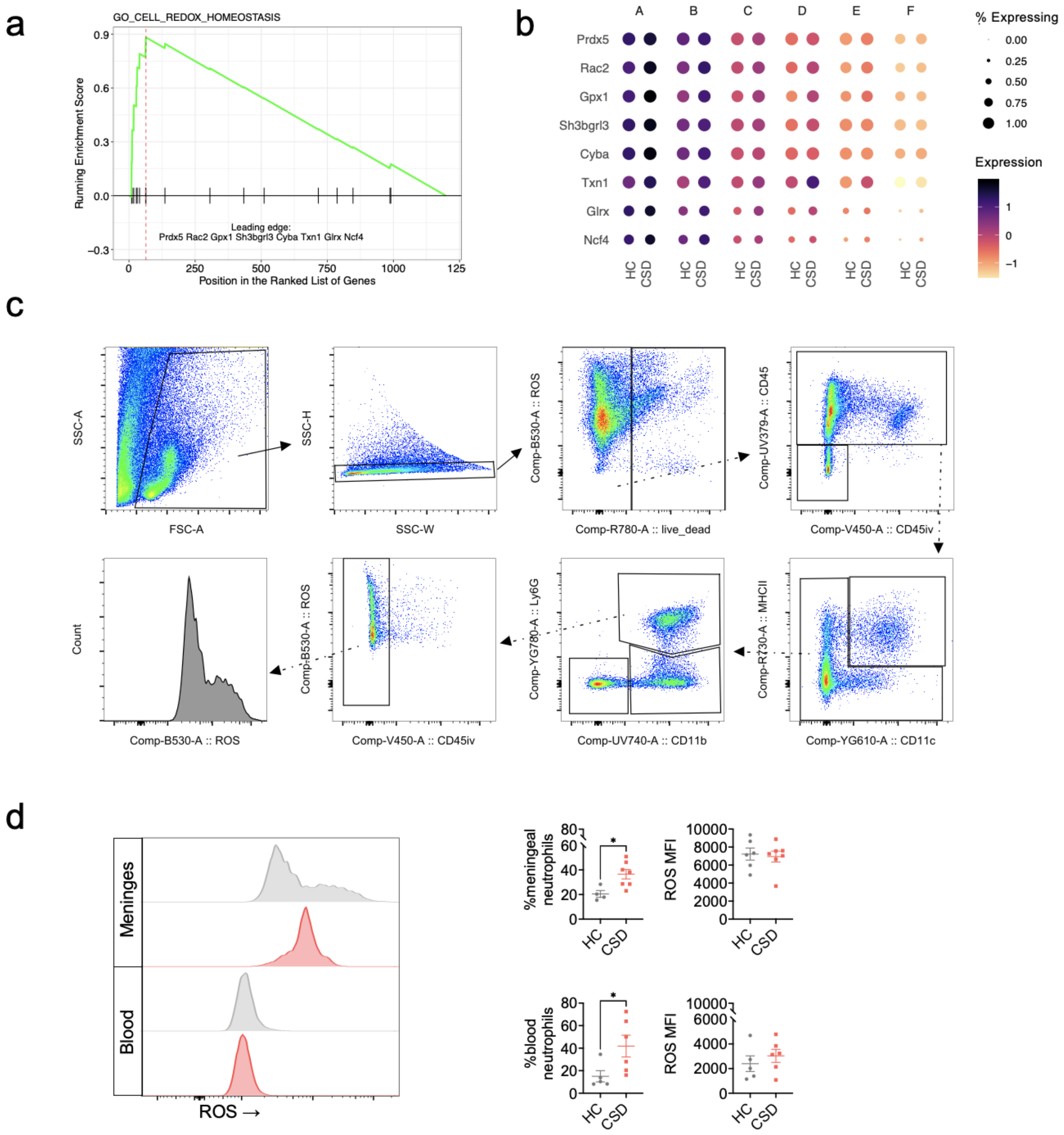

Figure S18: Response to reactive oxygen species (ROS)-based signaling in meningeal and blood neutrophils. **a**) Escarpment plot showing enrichment of GO: Cell redox homeostasis pathway in meningeal neutrophils; leading edge genes are listed as overlay. *Rac2, Cyba* and *Ncf4* are involved in formation of the phagocyte oxidase (phox) complex, which is in turn responsible for neutrophils' well-characterized ability to produce large amounts of reactive oxygen species (ROS) as an innate immune system defense mechanism[26]. **b**) Dot plot showing gene expression of leading-edge genes in each neutrophil subcluster for all genes comprising the GO:Cell redox homeostasis pathway. Expression is scaled to mean±standard deviation **c**) An intracellular ROS-labeling kit for flow cytometry was used to assess whether increased transcription of phox complex components was associated with increased ROS production in CSD meningeal neutrophils; gating strategy shown here. **d**) On a per-cell basis, there was no

difference in ROS production. This may stem from the counterbalancing effect of concomitantly increased antioxidant gene expression – including *Prdx5*, *Gpx1*, and *Txn1* and *Glrx*–in CSD meningeal neutrophils *Left*: Histograms showing representative intensity of ROS^+ cell staining in meningeal (*top*) and blood (*bottom*) neutrophils. *Right:* Validation of increased meningeal (*Top*; Unpaired t test: *p=0.019, t=2.9, df=9, n_{HC}=4, n_{CSD}=7) and blood (*Bottom*; Unpaired t test: *p=0.046, t=2.3, df=9, n_{HC}=5, n_{CSD}=6) neutrophils for animals in this study. No difference in ROS^+ staining between HC and CSD stress neutrophils (NB: there were three cohorts of mice included in this analysis. There was a large difference in average ROS MFIs between cohort 1 and cohorts 2 & 3, so a cohort average was generated for cohort 1 and cohorts 2 & 3, and a ratio of the two used to adjust cohort 1 values). The data generated in this experiment are included in the data presented in **Figures 1F-H** and **6G**; removal of these data does not affect the significant difference between groups shown in the figure. Tests were two-tailed. Data points in (**d**) represent individual mice. HC=home cage, CSD=chronic social defeat stress, MFI=median fluorescence intensity. Data shown as mean±SEM. Source data are provided as a Supplemental Source Data file.

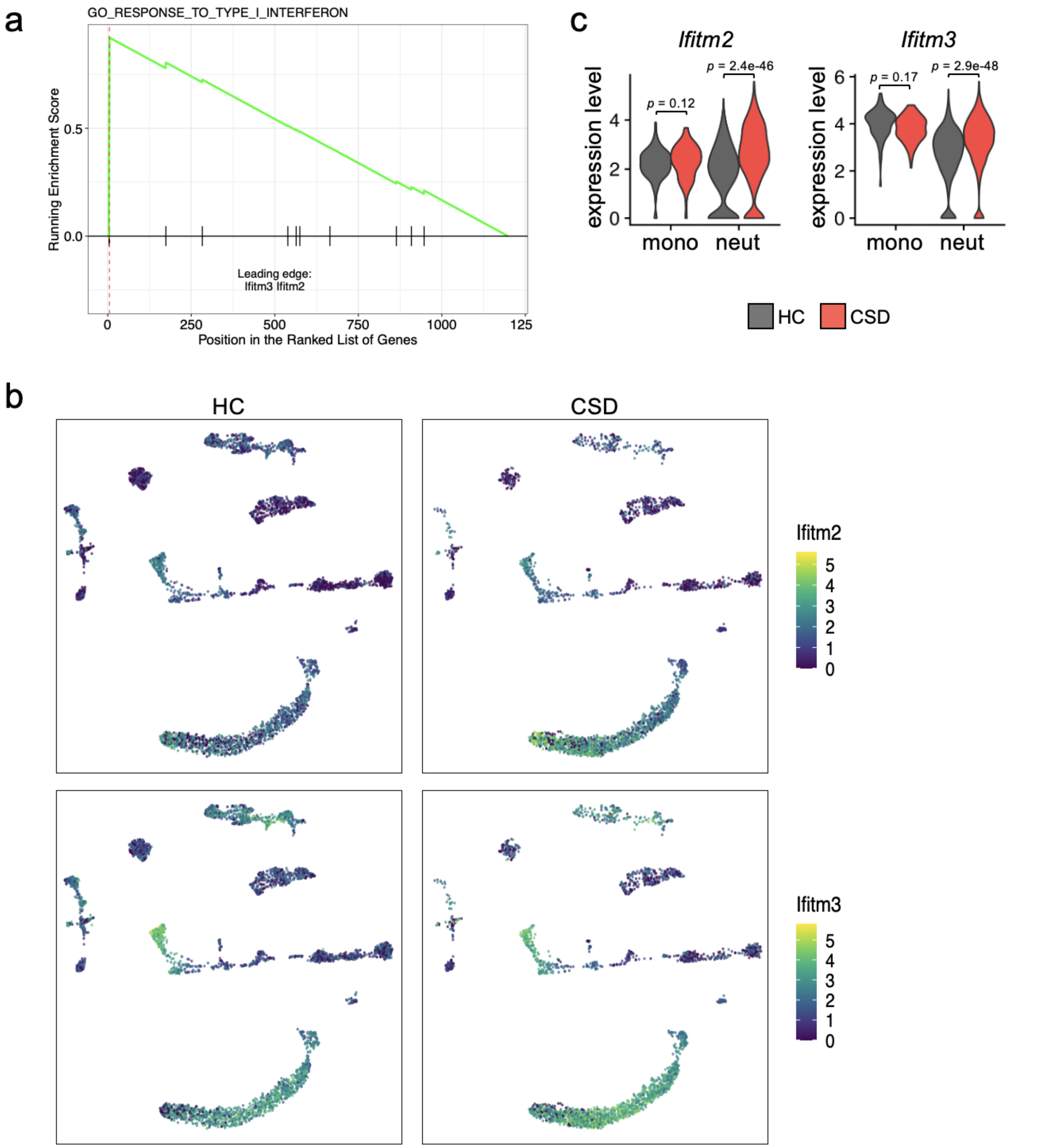

Figure S19: Increased type I interferon signaling following CSD stress is unique to meningeal neutrophils. **a**) *Top:* Escarpment plot showing enrichment of GO: Response to type I interferon pathway in meningeal neutrophils in stress; leading-edge genes are listed as overlay. *Bottom*: Increased expression of *Ifitm2* and *Ifitm3* in CSD meningeal neutrophils. **b**) Uniform manifold approximation and projection (UMAP) showing *Ifitm2* and *Ifitm3* expression in all meningeal cells for HC vs CSD mice. A subset of these data (specifically, the neutrophil cluster alone) are also presented in **Figure 7A. c**) While monocytes highly express both *Ifitm2* and *Ifitm3,* expression levels do not change with CSD stress. Data points represent individual cells from pooled sample. HC = home cage, CSD = chronic social defeat stress.

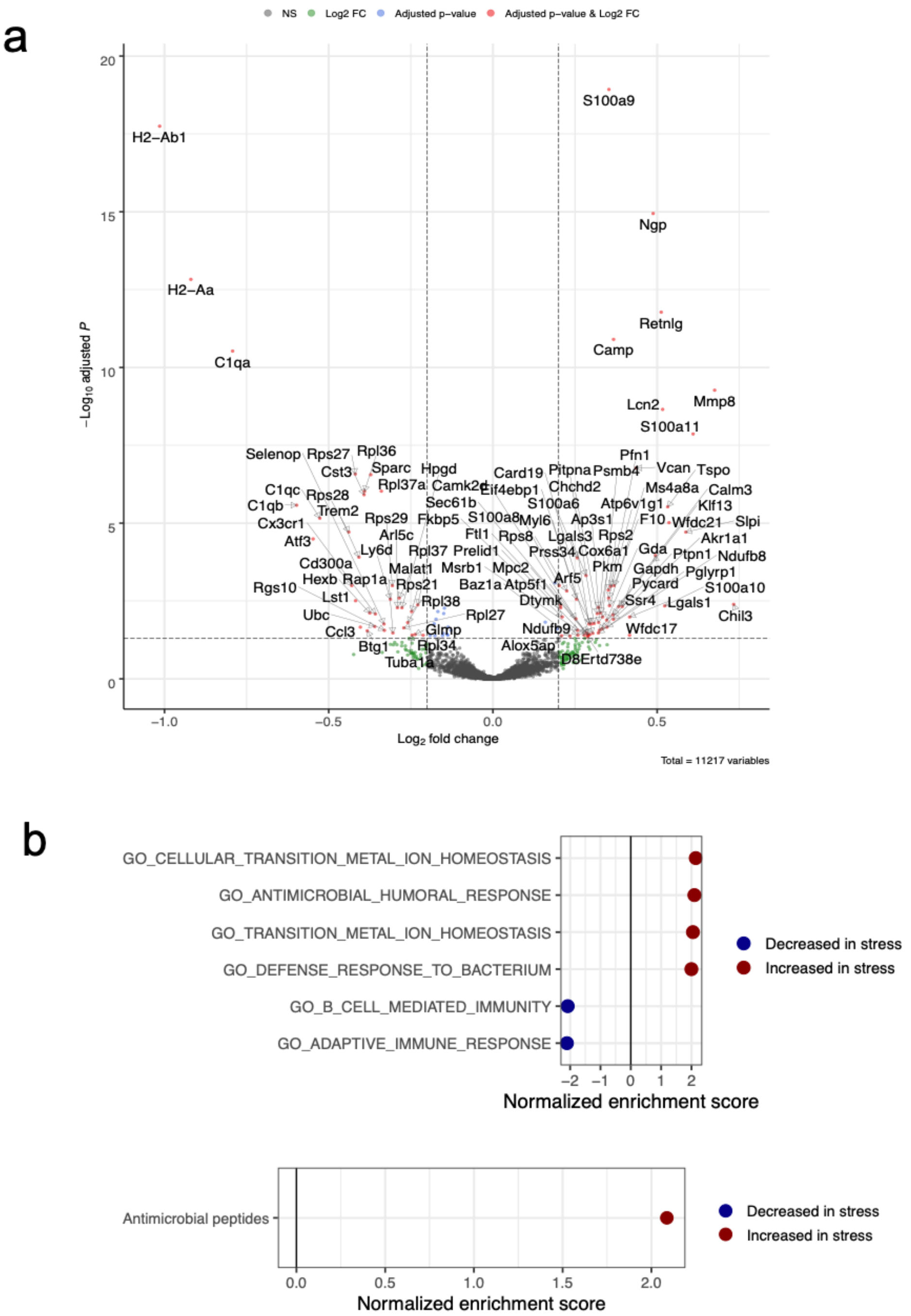

Figure S20: Meningeal monocytes do not show CSD-associated enrichment of interferon signaling. **a**) Volcano plot showing differentially expressed genes (DGE) between CSD and HC in the single cell RNAseq monocyte cluster. Indicated points represent DGE with LFC > 0.2 and FDR $p < 0.01$. Compare with neutrophil DGE in **Figure 6D**. **b**) Gene set enrichment analysis (GSEA) for biological pathways (top) and reactome (bottom). Compare with neutrophil GSEA in **Figure 6F**. CSD = chronic social defeat, HC = home cage, LFC = log fold change.

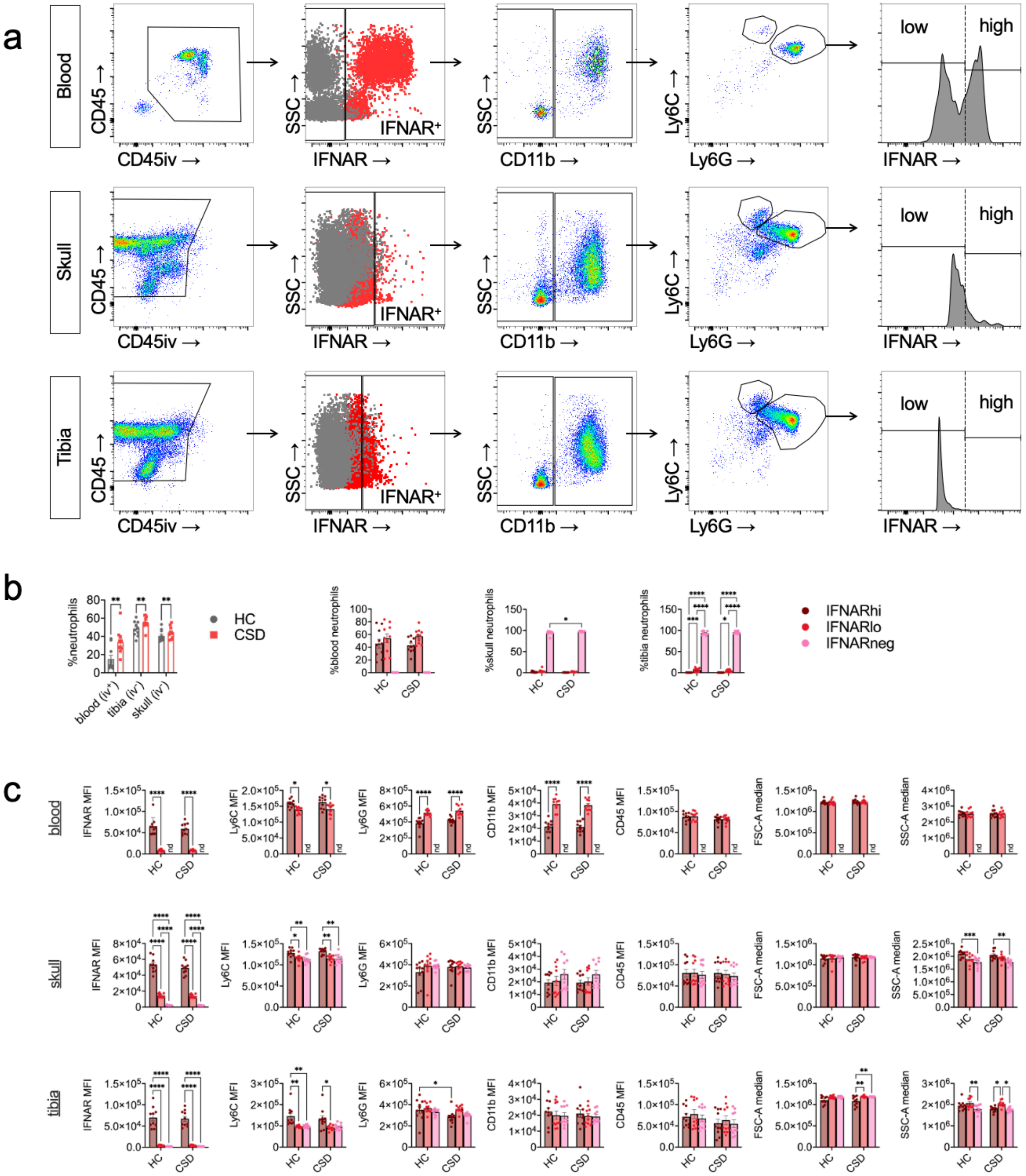

Figure S21: IFNAR^{+} neutrophil staining in peripheral immune tissues; data from these mice are also shown in **Figure 7D**. **a**) Gating strategy for assessing IFNAR^{+} neutrophil staining; a fluorescence minus one (FMO, shown in dark gray) control was used to set the negative gates for each tissue on each day of data acquisition (red = a representative stained sample). A CD45iv injection was used to discriminate bone marrow from vascular populations. Right-most histogram panel shows division of neutrophils into IFNAR^{hi} and IFNAR^{lo} populations. **b**) Elevated neutrophil levels in blood, skull, and tibia following CSD stress (group: ***p=0.0003, $F_{(1,56)}$=14. post-hoc: t=3.8, **p=0.0010). In skull, IFNAR^{neg} neutrophils increased with CSD stress

(group x cell type interaction, ***p*=0.0057, $F_{(2,54)}$=5.7. post-hoc: *t*=2.7, **p*=0.26). Tibia showed a main effect of cell type, but no group or interaction effect. **c**) Examination of median fluorescent intensities (MFIs), with groups separated into $IFNAR^{hi}$, $IFNAR^{lo}$, and $IFNAR^{neg}$ populations. *Blood*: CD11b MFI is lower in $IFNAR^{hi}$ vs $IFNAR^{lo}$ neutrophils (cell type: *****p*<0.0001, $F_{(1,36)}$=132). Ly6G MFI is lower in $IFNAR^{hi}$ vs $IFNAR^{lo}$ neutrophils (cell type: *****p*<0.0001, $F_{(1,36)}$=55.9), and higher in CSD compared to HC (group: **p*=0.050, $F_{(1,36)}$=4.12). Ly6C MFI is highest in $IFNAR^{hi}$ neutrophils (cell type: *****p*<0.0001, $F_{(1,36)}$=19.8). *Skull:* Ly6C MFI is highest in $IFNAR^{hi}$ neutrophils (cell type: *****p*<0.0001, $F_{(2,54)}$=19.7). SSC shows cell type-specific effect (cell type: *****p*<0.0001, $F_{(2,54)}$=17.0). Post-hoc analysis showed differences suggesting $IFNAR^{hi}$ populations have more granularity and/or complex nuclear morphology, consistent with $IFNAR^{hi}$ neutrophils being more mature than $IFNAR^{neg}$ neutrophils. *Tibia:* Ly6G MFI shows cell type-specific and CSD-related changes (group: **p*=0.014, $F_{(1,54)}$=6.5; cell type: **p*= 0.014, $F_{(2,54)}$=4.7); post-hoc analysis reveals CSD-related decrease specific to $IFNAR^{hi}$ cells (*t*=2.7, **p*=0.027). Ly6C MFI is highest in $IFNAR^{hi}$ neutrophils (cell type: *****p*<0.0001, $F_{(2,54)}$=16.2). FSC shows type-specific effect (cell type: *****p*<0.0001, $F_{(2,54)}$=13.8); post-hoc analysis revealed a CSD-specific decrease in cell size between $IFNAR^{lo}$/$IFNAR^{neg}$ and $IFNAR^{hi}$ neutrophils. This is consistent with $IFNAR^{hi}$ neutrophils representing a more mature phenotype[27]. SSC shows cell type-specific effect (cell type: *****p*<0.0001, $F_{(2,54)}$=12.8). Post-hoc analysis showed differences suggesting $IFNAR^{+}$ populations have more granularity and/or complex nuclear morphology, consistent with $IFNAR^{+}$ neutrophils being more mature than $IFNAR^{neg}$ neutrophils. For all tests, 2-way ANOVAs with Šídák's multiple comparions test were used. n_{HC} = 10, n_{CSD} = 10; data points in (**b,c**) represent individual mice. HC = home cage, CSD = chronic social defeat stress, FSC = forward scatter, SSC = side scatter. Data shown as mean ± SEM. Source data are provided as a Supplemental Source Data file.

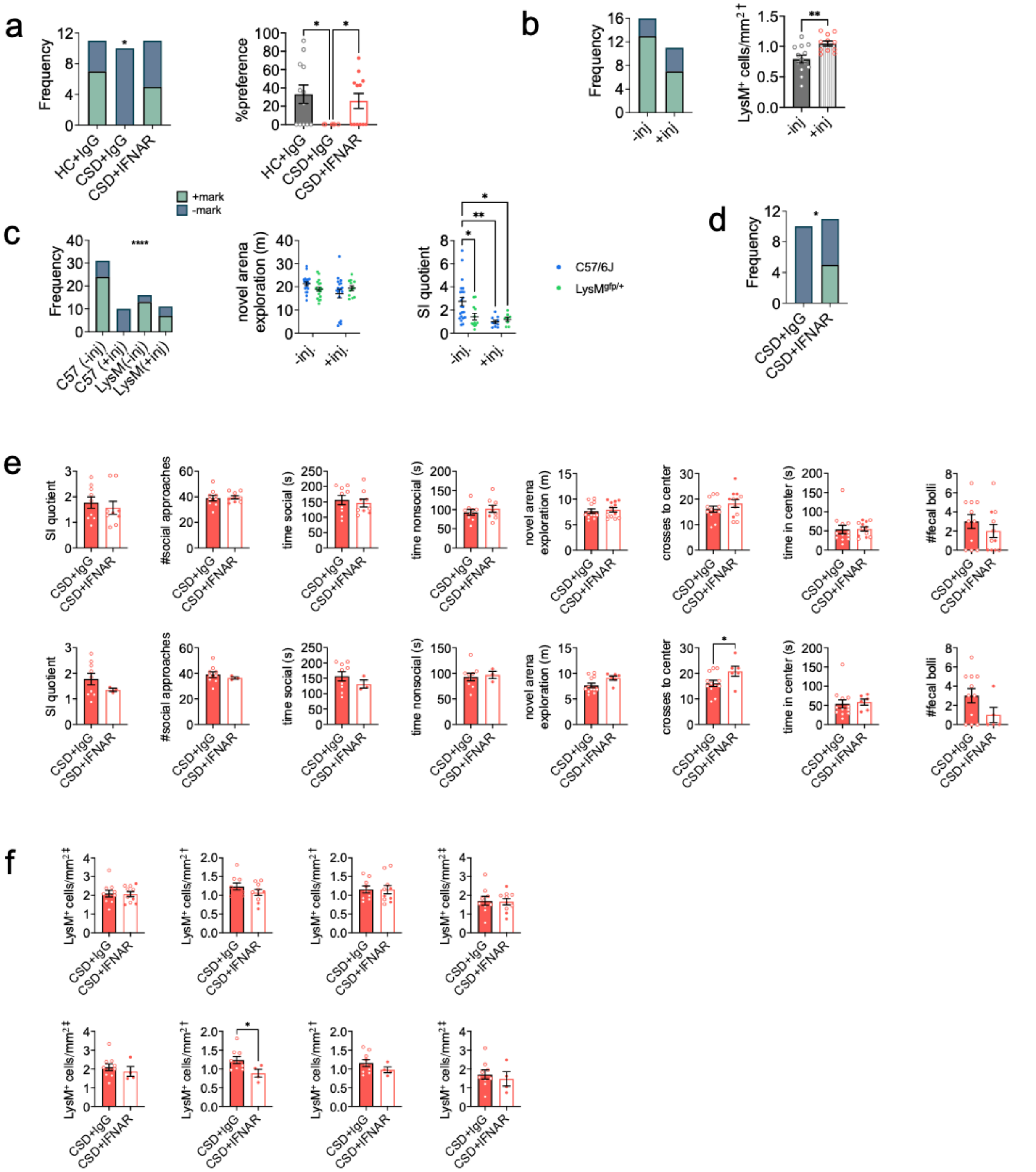

Figure S22: Anti-IFNAR treatment in LysM$^{gfp/+}$ mice improves anhedonic behavior in the USM task, but LysM$^{gfp/+}$ mice show strain-related effects of injection stress. **a**) Anti-IFNAR treatment protects against the anhedonic effects of stress. Control (IgG)-antibody treated mice exposed to CSD show expected behavioral deficits compared to HC-IgG mice. *Left*: marking behavior (χ^2 test, ***p*=0.0086, χ^2=9.5, *df*=2). *Right:* preference for female scent (Kruskal-Wallis test: ***P*=0.0088, *H*=9.5; Dunn's post-hoc: *$p_{HC\text{-}IgGvCSD\text{-}IgG}$=0.013, $Z_{HC\text{-}IgGvCSD\text{-}IgG}$=2.9; *$p_{CSD\text{-}IgGvCSD\text{-}IFNAR}$=0.048, $Z_{CSD\text{-}IgGvCSD\text{-}IFNAR}$=2.4; n=12 per group). **b**) *Left:* No effect of injection stress on USM behavior for HC LysM$^{gfp/+}$ mice (Fisher's exact text: *p*=0.39; n_{-inj}=16, n_{+inj}=11). *Right*: however,

there was an increase in nonvascular, meningeal LysM-GFP[+] cells with injection stress (**p=0.0040, t=3.2, df=22). **c**) Comparison of the effects of injection stress on strains. *Left*: Visualization of the percentage of either WT or LysM$^{gfp/+}$ mice that marked in the USM test; all animals shown here at HC. At baseline it appears that LysM$^{gfp/+}$ mice are more likely to mark than C57BL/6J mice (81% vs 62%). HC+IgG mice from both strains marked less frequently (64% of LysM$^{gfp/+}$ mice vs 0% of C57BL/6J mice); this was not a significant difference in LysM$^{gfp/+}$ mice (Fisher's exact text: p=0.39. *C57:* n_{-inj}=34, n_{+inj}=10; *LysM:* n_{-inj}=16, n_{+inj}=11). *Middle:* OF behavior not impacted by strain or injection status. *Right*: Significant interaction and status effects on the social interaction test; more anhedonic behavior in both strains with injection stress (2-way ANOVA test: interaction, *P=0.046, $F_{(1,48)}$=4.2; injection, *P=0.011, $F_{(1,48)}$=7.0. Tukey's posthoc: $C57_{-inj}$vs$LysM_{-inj}$ *p =0.027, q=4.1; $C57_{-inj}$vs$C57_{+inj}$ **p=0.0033, q=5.2; $C57_{-inj}$vs$LysM_{+inj}$ *p=0.024, q=4.2. *C57:* n_{-inj}=22, n_{+inj}=10; *LysM:* n_{-inj}=12, n_{+inj}=8). **d**) same data presented in **Figure 8B**, but binarized (Fisher's exact text: *p=0.035; n_{IgG}=10, n_{IFNAR}=11). **e**) Anhedonic (SI) and anxiety-like (OF) behavior in LysM$^{gfp/+}$ mice that underwent CSD stress. The top line shows all behavior patterns for all CSD+anti-IFNAR treated mice. The bottom line shows the same, but only shows CSD+anti-IFNAR treated mice that marked in the USM task. Only OF crosses to center were improved by anti-IFNAR treatment; these data are included in **Figure 8C** and are shown here for comparison. SI: n_{IgG}=9, n_{IFNAR}=9 (all) or 3 (+USM). OF: n_{IgG}=12, n_{IFNAR}=12 (all) or 6 (+USM). **f**) Reduced nonvascular (>10µm from blood vessel) meningeal LysM-GFP+ cells in LysM$^{gfp/+}$ mice that underwent CSD stress but received anti-IFNAR treatment. The top line shows meningeal handcount data for all CSD+anti-IFNAR treated mice. The bottom line shows the same but only CSD+anti-IFNAR treated mice that marked in the USM task are included. These data are included in **Figures 8D-E** and are shown here for comparison. No other meningeal subpopulations were significant. *Left to right*: total, nonvascular (>10µm from blood vessel), abluminal (≤10µm away from a blood vessel), and intravascular meningeal LysM-GFP+ cells. n_{IgG}=9, n_{IFNAR}=10 (all) or 4 (+USM). †square root-transformed, ‡ natural log-transformed values to improve normality. Univariate tests were two-tailed. Data points represent individual mice. Data shown as mean±SEM. Source data are provided as a Supplemental Source Data file.

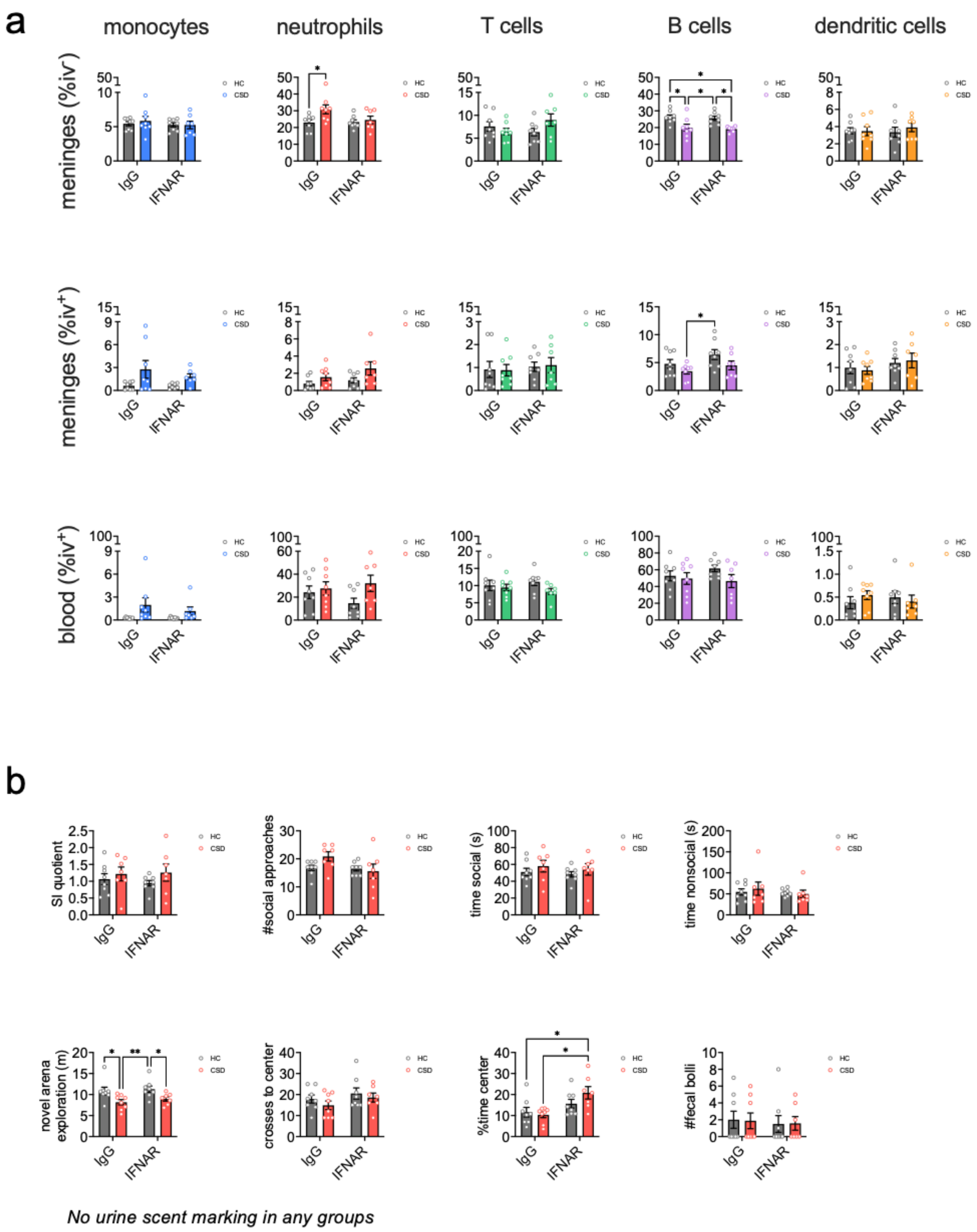

Figure S23: C57BL/6J mice do not show behavioral rescue with anti-IFNAR treatment, despite meningeal 'rescue' of neutrophils. **a**) Neutrophil data included for reference; see **Figure 8F.** *Top:* iv$^-$ meningeal cells, normalized to live, CD45$^+$. There was a trend for an interaction between anti-IFNAR and group in T cells (interaction, *P*=0.066, $F_{(1,27)}$=3.7), but no post-hoc significance. There was a suppressing effect of CSD on B cells independently of anti-IFNAR

treatment (main effect of group, ***P=0.0002, $F_{(1,27)}$=18.7. Tukey's post-hoc: *$p_{IgG\text{-}HCvIgG\text{-}CSD}$=0.023, $q_{IgG\text{-}HCvIgG\text{-}CSD}$=4.3; *$p_{IgG\text{-}HCvIFNAR\text{-}CSD}$=0.012, $q_{IgG\text{-}HCvIFNAR\text{-}CSD}$=4.8; *$p_{IgG\text{-}CSDvIFNAR\text{-}HC}$=0.049, $q_{IgG\text{-}CSDvIFNAR\text{-}HC}$=3.9; *$p_{IFNAR\text{-}HCvIFNAR\text{-}CSD}$=0.025, $q_{IFNAR\text{-}HCvIFNAR\text{-}CSD}$=4.3). *Middle:* iv$^+$ meningeal cells, normalized to live, CD45$^+$. For monocytes, CSD increased levels (main effect of group, *P=0.013, $F_{(1,27)}$=7.2), but no post-hoc significance. There was a suppressing effect of CSD on B cells independently of IFNAR treatment (main effect of group, *P=0.039, $F_{(1,27)}$=4.7. Tukey's post-hoc: *$p_{IgG\text{-}CSDvIFNAR\text{-}HC}$=0.039, $q_{IgG\text{-}CSDvIFNAR\text{-}HC}$=4.0). *Bottom*: blood cells, normalized to live, CD45$^+$. For monocytes, CSD increased levels (main effect of group, *P=0.025, $F_{(1,27)}$=5.7), but no post-hoc significance. **b**) *Top:* Social interaction (SI). There was a trend for an effect of anti-IFNAR treatment on social approaches (main effect of treatment, P=0.091, $F_{(1,26)}$=3.1), though nothing approached significance in post-hoc testing. No other statistical tests approached significance for SI. *Bottom*: Open Field (OF). There was a significant effect of anti-IFNAR treatment on OF time in center, where in both HC and CSD groups, time in the center increased, indicative of less anxious-like behavior (main effect of treatment, **P=0.0025, $F_{(1,27)}$=11.1. Tukey's post-hoc: *$p_{IgG\text{-}HCvIFNAR\text{-}CSD}$=0.032, $q_{IgG\text{-}HCvIFNAR\text{-}CSD}$=4.2; *$p_{IgG\text{-}CSDvIFNAR\text{-}CSD}$=0.012, $q_{IgG\text{-}CSDvIFNAR\text{-}CSD}$=4.7). IFNAR treatment had no improving effect on novel arena exploration; both CSD groups explored less than the HC groups (main effect of group, **P=0.0020, $F_{(1,27)}$=11.7. Tukey's post-hoc: *$p_{IgG\text{-}HCvIgG\text{-}CSD}$=0.015, $q_{IgG\text{-}HCvIgG\text{-}CSD}$=2.6; **$p_{IgG\text{-}CSDvIFNAR\text{-}HC}$=0.0050, $q_{IgG\text{-}CSDvIFNAR\text{-}HC}$=3.1; *$p_{IFNAR\text{-}HCvIFNAR\text{-}CSD}$=0.035, $q_{IFNAR\text{-}HCvIFNAR\text{-}CSD}$=2.2). For all experiments: $n_{IFNAR\text{-}CSD}$=7, otherwise n=8 per group. Data points represent individual mice. All tests were 2-way ANOVA with Tukey's post-hoc, corrected for multiple comparisons. Data shown as mean±SEM. Data were collected using the gating strategy shown in **Figures S2A-B** on samples from the mice shown in **Figures 8F**. HC=home cage, CSD=chronic social defeat stress, IFNAR = interferon-α/β receptor, SI = social interaction. Source data are provided as a Supplemental Source Data file.

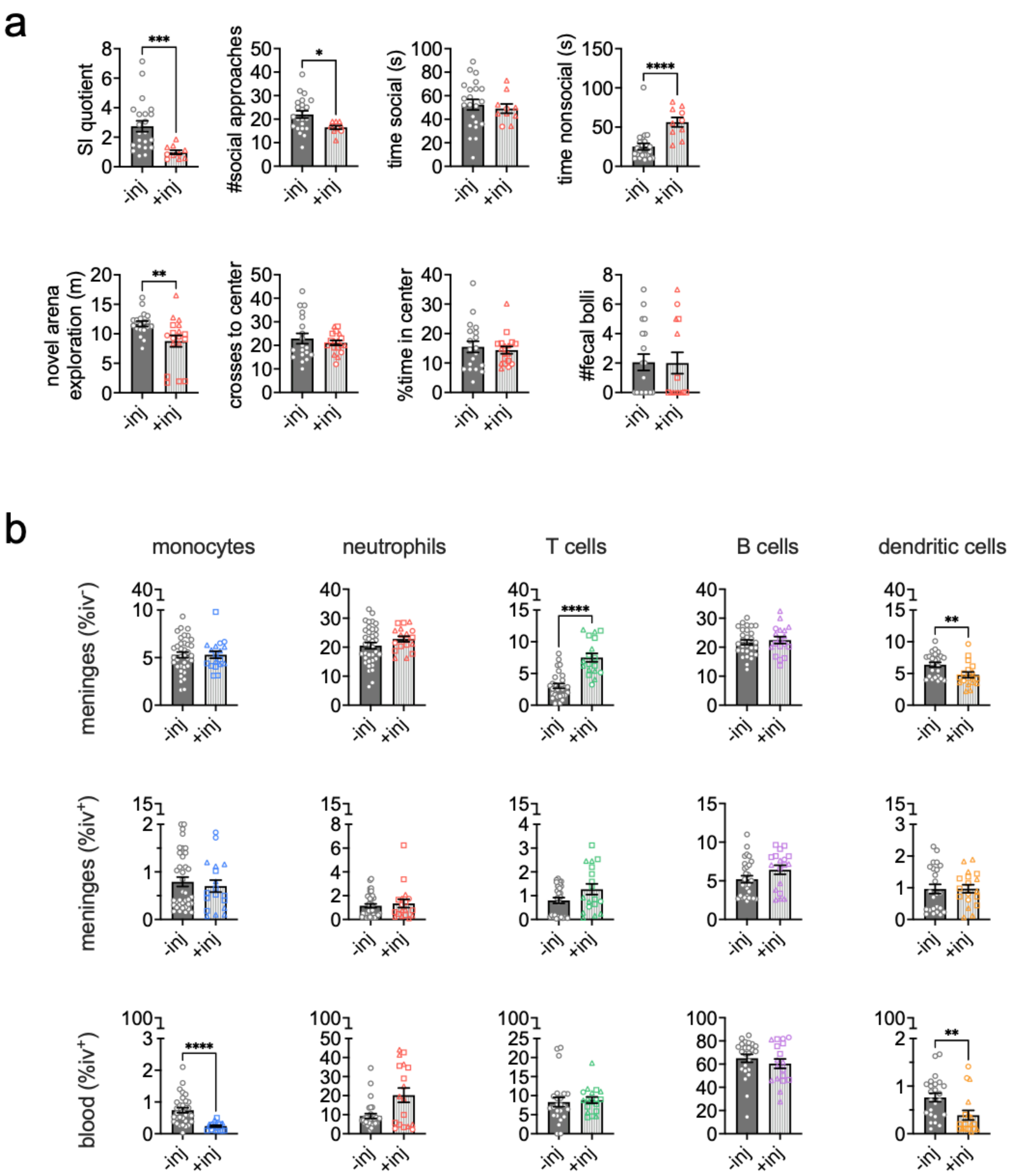

Figure S24: Exploration of the effect of injection and strain on behavior and immune cell dynamics in C57BL/6J home cage (HC) mice. **a**) *Top:* Social interaction. Repeated injections had an anhedonic effect on C57BL/6J mice for the SI quotient (***p = 0.0005, U = 29. n_{-inj} = 22, n_{+inj} = 10). The number of social approaches was also reduced (*p = 0.024, U = 55). Time spent interacting with the non-social object increased (****p < 0.0001, U = 21). *Bottom*: Open field. Repeated injections had an anxiogenic effect on C57BL/6J for exploration of a novel arena (**p = 0.010, U = 87. n_{-inj} = 19, n_{+inj} = 18). **b**) n C57BL/6J mice, there were no effects of injection stress on neutrophils. However, injection stress increased the percentage of iv^- meningeal T

cells (****p < 0.0001, U = 48.5. n_{-inj} = 29, n_{+inj} = 18) and decreased the percentage of iv^- meningeal dendritic cells (**p = 0.0075, U = 112. n_{-inj} = 24, n_{+inj} = 18). There were no effects on iv^+ meningeal cells. In blood, monocyte levels were decreased by injection stress (****p < 0.0001, U = 64. n_{-inj} = 32, n_{+inj} = 18). Neutrophil levels were not significantly elevated. Dendritic cell levels were reduced (**p = 0.0031, U = 102. n_{-inj} = 24, n_{+inj} = 18). Data points represent individual mice. All tests were two-tailed, Mann Whitney. Data shown as mean ± SEM. HC = home cage, CSD = chronic social defeat. Inj = injection, SI = social interaction. Source data are provided as a Supplemental Source Data file.

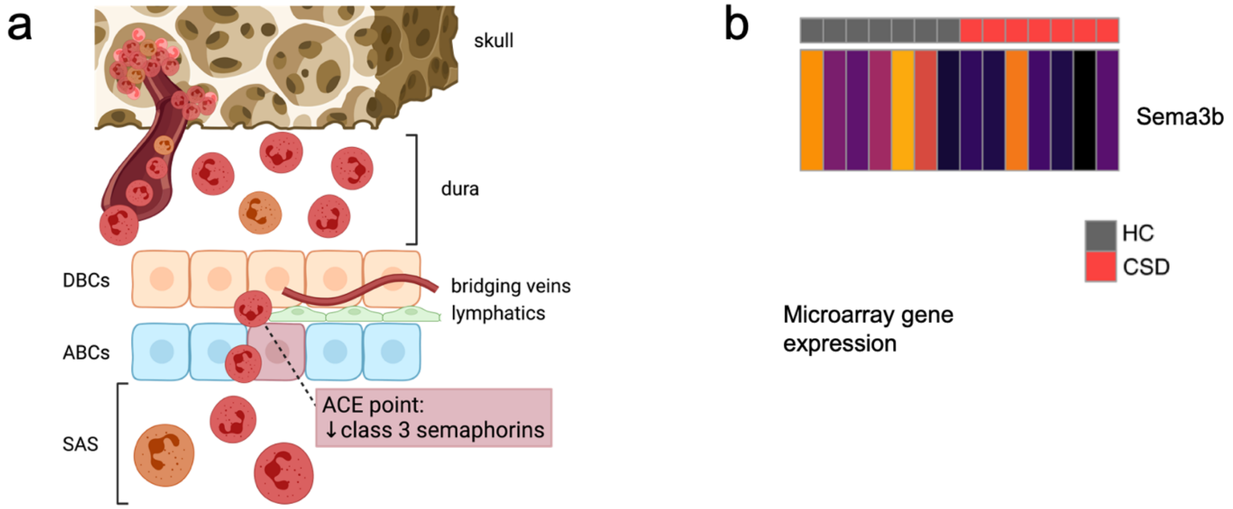

Figure S25: Potential mechanisms of neutrophil trafficking to the meninges and neutrophil-brain communication. **a**) Class 3 semaphorins (SEMA3) have chemorepellant properties that prevent migration of neutrophils across dural lymphatics into the leptomeninges at arachnoid cuff exit (ACE) points[11]. Depletion of SEMA3 family expression leads to accumulation of neutrophils in subarachnoid space (SAS) where neutrophil-released factors could influence brain function. **b**) Microarray analysis of SEMA3 family members in bulk meningeal tissue. *Sema3b* expression was reduced in CSD mice compared to HC (LFC = -0.30, *unadjusted *P* = 0.050), which may permit neutrophil entry into the SAS (n = 7 per group). Communication between meningeal neutrophils and brain-resident cells may occur via direct cell–cell contact or through the secretion of bioactive molecules that influence neural, endothelial, or glial function. Notably, CSD meningeal neutrophils exhibited broad upregulation of secreted factors with potential neuroimmune activity, including *Lgals3* (Galectin-3), the neuroprotective factor *Slpi*, the alarmins *S100a8/S100a9*, and *Lcn2*. ABC = arachnoid barrier cell, CSD = chronic social defeat, DBC = dural border cell, HC = home cage. Schematic created in BioRender. Kigar, S. (2025) https://BioRender.com/b28h656

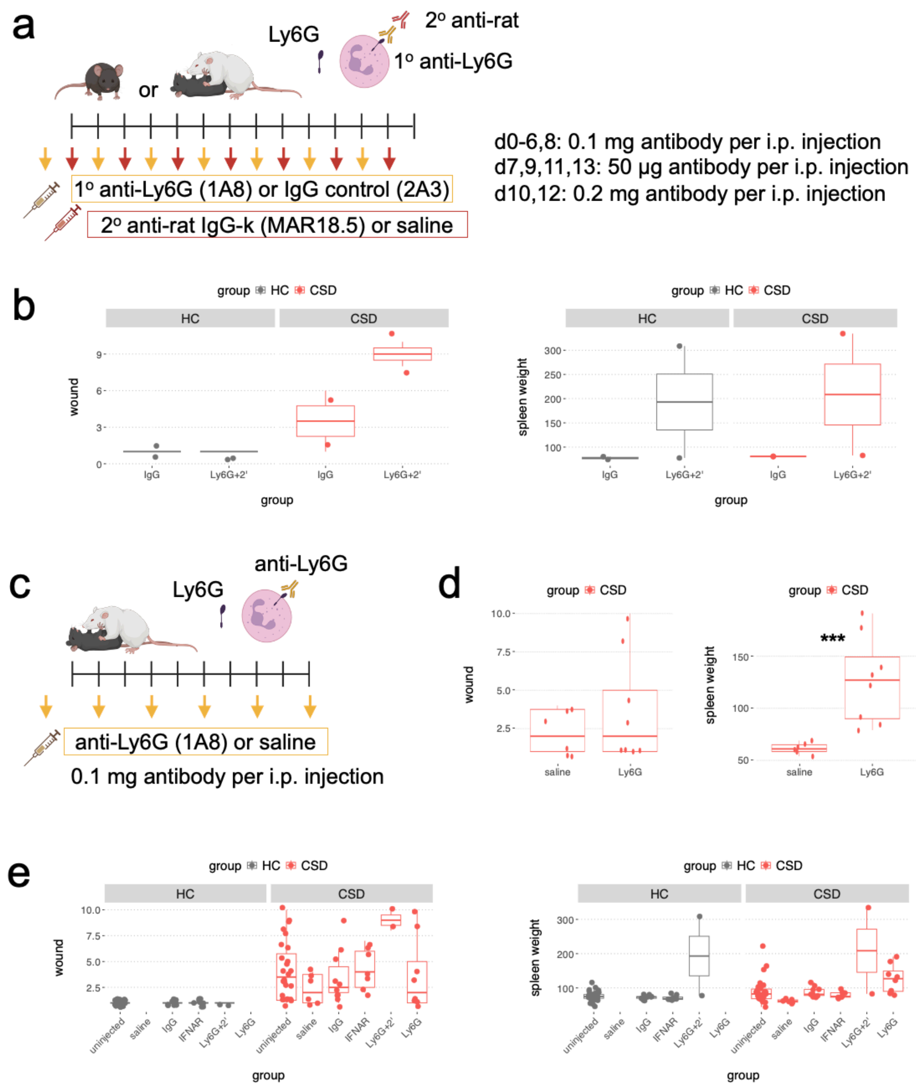

Figure S26: Systemic neutrophil depletion is poorly compatible with CSD stress. **a**) Neutrophil depletion paradigm illustrating schedule of anti-Ly6G and secondary anti-rat injections, based on previously published methods[28]. The 4 experimental groups were HC+IgG (control), HC+Ly6G, CSD+IgG (control) and CSD+Ly6G (n = 2 per group). **b**) Wound scores and spleen weights for mice used in (**a**). Massive splenomegaly present in some animals, combined with informal observations that ear punches to track individuals did not heal properly—and on the contrary, expanded—and the concerning appearance of wound injuries in CSD mice led to early termination of the study. **c**) Modified neutrophil depletion paradigm illustrating reduced number of defeat encounters. All mice were exposed to defeat; saline injected mice served as a control (n_{saline} = 6, n_{Ly6G} = 8). **d**) Wound scores and spleen weights for mice used in (**c**). There was no difference in wound severity with the modified paradigm, however splenomegaly was significantly increased in Ly6G-treated mice compared to saline (Welch's t-test: **p = 0.0028, t = -4.4, df = 7.3). **e**) Wound scores and spleen weights for both Ly6G studies—Ly6G+2' (**a**) or Ly6G alone (**c**)—are shown in comparison to mice in other studies shown throughout the manuscript. Data points represent individual mice. HC=home cage, CSD=chronic social defeat stress, IFNAR = interferon-α/β receptor, Source data are provided as a Supplemental Source Data file. Schematics created in BioRender. Kigar, S. (2025) https://BioRender.com/rvrmii4.

Supplemental References:

1. Lynall, M.-E. *et al.* B-cells are abnormal in psychosocial stress and regulate meningeal myeloid cell activation. *Brain. Behav. Immun.* **97**, 226–238 (2021).
2. Burns, A. R. *et al.* P-selectin mediates neutrophil adhesion to endothelial cell borders. *J. Leukoc. Biol.* **65**, 299–306 (1999).
3. Hodes, G. E. *et al.* Sex Differences in Nucleus Accumbens Transcriptome Profiles Associated with Susceptibility versus Resilience to Subchronic Variable Stress. *J. Neurosci.* **35**, 16362–16376 (2015).
4. Menard, C. *et al.* Social stress induces neurovascular pathology promoting depression. *Nat. Neurosci.* **20**, 1752–1760 (2017).
5. Dion-Albert, L. *et al.* Vascular and blood-brain barrier-related changes underlie stress responses and resilience in female mice and depression in human tissue. *Nat. Commun.* **13**, 164 (2022).
6. Stadtmann, A. & Zarbock, A. CXCR2: From Bench to Bedside. *Front. Immunol.* **3**, (2012).
7. Evrard, M. *et al.* Developmental Analysis of Bone Marrow Neutrophils Reveals Populations Specialized in Expansion, Trafficking, and Effector Functions. *Immunity* **48**, 364-379.e8 (2018).
8. Herisson, F. *et al.* Direct vascular channels connect skull bone marrow and the brain surface enabling myeloid cell migration. *Nat. Neurosci.* **21**, 14 (2018).
9. Cugurra, A. *et al.* Skull and vertebral bone marrow are myeloid cell reservoirs for the meninges and CNS parenchyma. *Science* eabf7844 (2021) doi:10.1126/science.abf7844.
10. Kolabas, Z. I. *et al.* Distinct molecular profiles of skull bone marrow in health and neurological disorders. *Cell* **186**, 3706-3725.e29 (2023).
11. Smyth, L. C. D. *et al.* Identification of direct connections between the dura and the brain. *Nature* **627**, 165–173 (2024).

12. Xie, X. *et al.* Single-cell transcriptome profiling reveals neutrophil heterogeneity in homeostasis and infection. *Nat. Immunol.* **21**, 1119–1133 (2020).
13. Grieshaber-Bouyer, R. *et al.* The neutrotime transcriptional signature defines a single continuum of neutrophils across biological compartments. *Nat. Commun.* **12**, 2856 (2021).
14. Goley, E. D. & Welch, M. D. The ARP2/3 complex: an actin nucleator comes of age. *Nat. Rev. Mol. Cell Biol.* **7**, 713–726 (2006).
15. Sun, C. X., Magalhães, M. A. O. & Glogauer, M. Rac1 and Rac2 differentially regulate actin free barbed end formation downstream of the fMLP receptor. *J. Cell Biol.* **179**, 239–245 (2007).
16. Scheller, I. *et al.* Thymosin β4 is essential for thrombus formation by controlling the G-actin/F-actin equilibrium in platelets. *Haematologica* **107**, 2846–2858 (2021).
17. Iwata, M. *et al.* Psychological Stress Activates the Inflammasome via Release of Adenosine Triphosphate and Stimulation of the Purinergic Type 2X7 Receptor. *Biol. Psychiatry* **80**, 12–22 (2016).
18. Martin, P. *et al.* Mouse neutrophils express the decoy type 2 interleukin-1 receptor (IL-1R2) constitutively and in acute inflammatory conditions. *J. Leukoc. Biol.* **94**, 791–802 (2013).
19. Kwok, A. J. *et al.* Neutrophils and emergency granulopoiesis drive immune suppression and an extreme response endotype during sepsis. *Nat. Immunol.* **24**, 767–779 (2023).
20. Liles, W. C., Kiener, P. A., Ledbetter, J. A., Aruffo, A. & Klebanoff, S. J. Differential expression of Fas (CD95) and Fas ligand on normal human phagocytes: implications for the regulation of apoptosis in neutrophils. *J. Exp. Med.* **184**, 429–440 (1996).
21. Steinhauser, M. L. *et al.* Chemokine C10 Promotes Disease Resolution and Survival in an Experimental Model of Bacterial Sepsis. *INFECT IMMUN* **68**, (2000).
22. Martinelli, S. *et al.* Induction of Genes Mediating Interferon-dependent Extracellular Trap Formation during Neutrophil Differentiation. *J. Biol. Chem.* **279**, 44123–44132 (2004).

23. Kerfoot, S. M. *et al.* Exclusive Neutrophil Recruitment with Oncostatin M in a Human System. *Am. J. Pathol.* **159**, 1531–1539 (2001).

24. Cross, A., Edwards, S. W., Bucknall, R. C. & Moots, R. J. Secretion of oncostatin M by neutrophils in rheumatoid arthritis. *Arthritis Rheum.* **50**, 1430–1436 (2004).

25. Heidt, T. *et al.* Chronic variable stress activates hematopoietic stem cells. *Nat. Med.* **20**, 754–758 (2014).

26. Nguyen, G. T., Green, E. R. & Mecsas, J. Neutrophils to the ROScue: Mechanisms of NADPH Oxidase Activation and Bacterial Resistance. *Front. Cell. Infect. Microbiol.* **7**, 373 (2017).

27. Lichtman, M. A. & Weed, R. I. Alteration of the Cell Periphery During Granulocyte Maturation: Relationship to Cell Function. *Blood* **39**, 301–316 (1972).

28. Faget, J. *et al.* Efficient and specific Ly6G$^+$ cell depletion: A change in the current practices toward more relevant functional analyses of neutrophils. Preprint at https://doi.org/10.1101/498881 (2018).
