# Supplementary material for: Chronic social defeat stress induces meningeal neutrophilia via type I interferon signaling in male mice
Source: Nat Commun. 2025 Sep 1;16:8153. doi: 10.1038/s41467-025-62840-5 (PMC12402070; doi:10.1038/s41467-025-62840-5)
Supplement: Supplementary file 2 — Reporting Summary [file 41467_2025_62840_MOESM2_ESM.pdf]

nature portfolio

Corresponding author(s): Stacey Kigar

Last updated by author(s): Jul 10, 2025

Reporting Summary

Nature Portfolio wishes to improve the reproducibility of the work that we publish. This form provides structure for consistency and transparency in reporting. For further information on Nature Portfolio policies, see our Editorial Policies and the Editorial Policy Checklist.

Statistics

For all statistical analyses, confirm that the following items are present in the figure legend, table legend, main text, or Methods section.

n/a	Confirmed	
☐	☒	The exact sample size (*n*) for each experimental group/condition, given as a discrete number and unit of measurement
☐	☒	A statement on whether measurements were taken from distinct samples or whether the same sample was measured repeatedly
☐	☒	The statistical test(s) used AND whether they are one- or two-sided<br>*Only common tests should be described solely by name; describe more complex techniques in the Methods section.*
☐	☒	A description of all covariates tested
☐	☒	A description of any assumptions or corrections, such as tests of normality and adjustment for multiple comparisons
☐	☒	A full description of the statistical parameters including central tendency (e.g. means) or other basic estimates (e.g. regression coefficient) AND variation (e.g. standard deviation) or associated estimates of uncertainty (e.g. confidence intervals)
☐	☒	For null hypothesis testing, the test statistic (e.g. *F*, *t*, *r*) with confidence intervals, effect sizes, degrees of freedom and *P* value noted<br>*Give P values as exact values whenever suitable.*
☒	☐	For Bayesian analysis, information on the choice of priors and Markov chain Monte Carlo settings
☐	☒	For hierarchical and complex designs, identification of the appropriate level for tests and full reporting of outcomes
☐	☒	Estimates of effect sizes (e.g. Cohen's *d*, Pearson's *r*), indicating how they were calculated

Our web collection on statistics for biologists contains articles on many of the points above.

Software and code

Policy information about availability of computer code

Data collection	Capture Star Version 1.00 (behavior), Zeiss ZEN (microscopy data), BD Diva (flow cytometry), native software for Beckman Coulter CytoFLEX (flow cytometry), Amnis IDEAS (for imaging flow cytometry), Cellranger V2 (single cell)
Data analysis	TopScan (behavior analysis, version unknown), ImageJ (urine scent marking and microscopy analysis, version unknown), FlowJo (flow cytometry data, several versions), IMARIS (cleared tissue data, version 9.7), Prism 9.0.2 and 10.0 (for univariate statistics), R (for multivariate statistics and single cell sequencing analysis; multiple versions). Bespoke R code with version notations can be accessed at https://github.com/staceykigar/meningeal_neut

For manuscripts utilizing custom algorithms or software that are central to the research but not yet described in published literature, software must be made available to editors and reviewers. We strongly encourage code deposition in a community repository (e.g. GitHub). See the Nature Portfolio guidelines for submitting code & software for further information.

Data

Policy information about [availability of data](#)

All manuscripts must include a [data availability statement](#). This statement should provide the following information, where applicable:

- Accession codes, unique identifiers, or web links for publicly available datasets
- A description of any restrictions on data availability
- For clinical datasets or third party data, please ensure that the statement adheres to our [policy](#)

Single cell sequencing data generated in this study have been deposited in the GEO database under accession code GSE301684 [https://www.ncbi.nlm.nih.gov/geo/query/acc.cgi?&acc=GSE301684]. Publicly available single cell sequencing data analyzed in this study are available online under accession codes GSE192616 [https://www.ncbi.nlm.nih.gov/geo/query/acc.cgi?acc=GSE192616] and GSE109467 [https://www.ncbi.nlm.nih.gov/geo/query/acc.cgi?acc=GSE109467]. Microarray data analyzed in this study have been deposited in the GEO database under accession code GSE275966 [https://www.ncbi.nlm.nih.gov/geo/query/acc.cgi?acc=GSE275966]. Other data generated in this study are provided in the Source Data and Supplementary Source Data files provided with this paper.

Research involving human participants, their data, or biological material

Policy information about studies with [human participants or human data](#). See also policy information about [sex, gender (identity/presentation), and sexual orientation](#) and [race, ethnicity and racism](#).

Reporting on sex and gender	n/a
Reporting on race, ethnicity, or other socially relevant groupings	n/a
Population characteristics	n/a
Recruitment	n/a
Ethics oversight	n/a

Note that full information on the approval of the study protocol must also be provided in the manuscript.

Field-specific reporting

Please select the one below that is the best fit for your research. If you are not sure, read the appropriate sections before making your selection.

☒ Life sciences ☐ Behavioural & social sciences ☐ Ecological, evolutionary & environmental sciences

For a reference copy of the document with all sections, see [nature.com/documents/nr-reporting-summary-flat.pdf](#)

Life sciences study design

All studies must disclose on these points even when the disclosure is negative.

Sample size	No power analyses were conducted prior to data collection. We used a similar sample size for flow cytometry data to that in our previously published data (10.1016/j.bbi.2021.08.002). We generally aimed for 8-12 animals per group for behavior studies as this is commonly reported in the literature.
Data exclusions	Animals were shaved to assess wounding at the end of the CSD paradigm; an internally validated reviewing system was developed to rate wound severity on a scale from 1-10 (1 = no wounds, 10 severe wounds). Animals with a wound score of 10 were excluded. Samples with suspected technical artifacts were excluded (e.g., on one dissection day, PBS was used instead of fixative for histology). One pooled sample of CSD mice for single cell sequencing was excluded as the droplet encapsulation step failed and the data quality was too poor for analysis.
Replication	The main finding of the paper (meningeal neutrophil counts elevated by CSD) were replicated in two strains (C57 and LysM) of mice using several techniques (flow, IHC, single cell sequencing results) in multiple experimental cohorts over a period of several years. The flow cytometry experiments were also replicated in several of our biological assays when attempting to bolster support for our single cell sequencing data. Behavioral findings were similar between C57 and LysM strains. We attempted to replicate our single cell sequencing results using biological assays; these data were consistent with our single cell data, except that we did not see an increase in CXCR2 protein on CSD meningeal neutrophils. This could represent differences in mRNA vs protein, or it could be a consequence of having a mixed population of neutrophils collapsed as one pool for flow. We also attempted to replicate the main rescue experiment of IFNAR blockade in both LysM and C57 mice. Control C57 mice were behaviorally sensitive to the effects of repeated injection stress, and none of the mice 'marked' in the USM test. LysM showed a mixed response to treatment in terms of both behavior and meningeal neutrophil levels. We emphasize the need for additional replication studies to conclusively establish this pathway in our discussion.
Randomization	Age- and sex-matched mice were randomly assigned to group.
Blinding	For behavioral data acquisition, blinding to group would not be possible while placing animals into the behavioral task arenas, as HC animals

Blinding

and CSD animals have different cage types. Thus, behavioral data are analyzed by TopScan animal-tracking software program to eliminate bias. At dissection, at least two people were present so that the person dissecting was handed a mouse and did not know what group the mouse came from. Mice were given a random ID number from that point forward so that all subsequent data analysis steps were conducted while blinded.

Reporting for specific materials, systems and methods

We require information from authors about some types of materials, experimental systems and methods used in many studies. Here, indicate whether each material, system or method listed is relevant to your study. If you are not sure if a list item applies to your research, read the appropriate section before selecting a response.

Materials & experimental systems

n/a	Involved in the study
☐	☒ Antibodies
☒	☐ Eukaryotic cell lines
☒	☐ Palaeontology and archaeology
☐	☒ Animals and other organisms
☒	☐ Clinical data
☒	☐ Dual use research of concern
☒	☐ Plants

Methods

n/a	Involved in the study
☒	☐ ChIP-seq
☐	☒ Flow cytometry
☒	☐ MRI-based neuroimaging

Antibodies

Antibodies used

multiple lots were used for all antibodies.

flow:
B220-BV711 RA3-6B2 Biolegend 103255 - 1.5 µL/rx
CD11b-APC/Fire750 M1/70 Biolegend 101262 – 2 µL/rx
CD11b-BUV737 M1/70 BD Biosciences 612800 - 2 µL/rx
CD11b-PE/Cy7 M1/70 Biolegend 101216 - 2 µL/rx
CD11b-PerCP/Cy5.5 M1/70 Biolegend 101228 - 2 µL/rx
CD11c-PE/Cy7 N418 Biolegend 117318 – 1.5 µL/rx
CD11c-PE/Dazzle594 N418 Biolegend 117348 - 1.5 µL/rx
CD19-BV711 6D5 Biolegend 115555 - 1.5 µL/rx
CD19-PE/Cy7 6D5 Biolegend 115520 - 1.5 µL/rx
CD206-BV650 C068C2 Biolegend 141723 – 2 µL/rx
CD3-AF488 17A2 Biolegend 100210 - 2 µL/rx
CD3-BV421 17A2 Biolegend 100228 - 2 µL/rx
CD3-PE 17A2 Biolegend 100206 - 2 µL/rx
CD3-PerCP/Cy5.5 17A2 Biolegend 100218 - 2 µL/rx
CD31-PE 390 Biolegend 102407 - 4 µL/rx
CD31-PE/Dazzle594 390 Biolegend 102430 - 4 µL/rx
CD4-BUV395 GK1.5 BD Biosciences 563790 - 1.5 µL/rx
CD4-BV421 RM4-5 Biolegend 100544 - 1.5 µL/rx
CD4-BV711 RM4-5 Biolegend 100550 - 1.5 µL/rx
CD44-APC IM7 Biolegend 103012 – 1.5 µL/rx
CD44-BV785 IM7 Biolegend 103059 – 1.5 µL/rx
CD44-PE IM7 Biolegend 103008 – 1.5 µL/rx
CD45-AF647 30-F11 Biolegend 103124 – 1.5 µL/rx
CD45-BUV395 30-F11 BD Biosciences 564279 1.5 µL/rx or 8µL if injected
CD45-BV421 30-F11 Biolegend 103134 - 1.5 µL/rx
CD45-FITC 30-F11 Biolegend 103108 - 1.5 µL/rx or 8µL if injected
CD62L-APC MEL-14 Biolegend 104412 1.5 µL/rx
CD62L-BUV737 MEL-14 BD Biosciences 565213 1.5 µL/rx
CD8-BV421 53-6.7 Biolegend 100738 - 1.5 µL/rx
CD8-BV510 53-6.7 Biolegend 100751 - 1.5 µL/rx
CD8-PE/Cy7 53-6.7 Biolegend 100722 - 1.5 µL/rx
CXCR2-APC SA044G4 Biolegend 149311 - 1 µL/rx
CXCR4-BV421 2B11 BD Biosciences 562738 - 1 µL/rx
ICAM-PE/Cy7 YN1/1.7.4 Biolegend 116121 - 1 µL/rx
IFNAR1-PE MAR1-5A3 Biolegend 127312- 5 µL/rx
Ly6C-BV650 HK1.4 Biolegend 128049 - 1 µL/rx
Ly6C-BV785 HK1.4 Biolegend 128041 - 1 µL/rx
Ly6C-PerCP/Cy5.5 HK1.4 Biolegend 128011 - 1 µL/rx
Ly6G-AF647 1A8 Biolegend 127610 - 1 µL/rx
Ly6G-APC 1A8 Biolegend 127614 - 1 µL/rx
Ly6G-BV510 1A8 Biolegend 127633 - 1 µL/rx
Ly6G-BV605 1A8 Biolegend 127639 - 1 µL/rx
Ly6G-PE/Cy7 1A8 Biolegend 127617 - 1 µL/rx

MHCI-BUV496 28-8-6 BD Biosciences 750133 - 1 µL/rx
MHCII-AF700 M5/114.15.2 ThermoFisher 56-5321-82 – 0.5 µL/rx
VCAM-APC 429 (MVCAM.A) Biolegend 105717 - 4 µL/rx

immunohistochemistry
chicken anti-GFP Abcam 13970 – 1:1000 dilution
Chicken IgY-Alexa Fluor 488 Abcam 150169 – 1:500 dilution
mouse p-Selectin Biolegend 148301 – 1:1000 dilution
Alexa Fluor 555 anti-mouse Invitrogen A-31570 – 1:500 dilution

In vivo injection
Mouse anti-mouse IFNAR MAR1-5A3 Bio X Cell BE0241 – 1mg or 0.5mg
Mouse IgG MOPC-21 Bio X Cell BE0083 – 1mg or 0.5mg
Rat anti-mouse Ly6G 1A8 Bio X Cell BE0075 – 0.2mg or 0.1mg
Rat anti-mouse IgG-κ MAR18.5 Bio X Cell BE0122 - 50µg or 0.1mg
Rat IgG 2A3 Bio X Cell BE0089 – 0.2mg or 0.1mg

Validation

All cell lineage flow cytometry antibodies were used as recommended by experts at NINDS and NCI; control mouse pilot experiments in blood showed relative cell percentages consistent with reported values (https://www.bio-rad-antibodies.com/static/2017/flow/flow-cytometry-cell-frequency.pdf). Activation markers (CXCR2, CXCR4, IFNAR, CD31, ICAM1, MHCI, MHCII, VCAM) for flow were verified with fluorescence-minus one (FMO) controls. FMO data for CXCR2, CXCR4, and IFNAR are shown in supplemental figures. Secondary-only pilot studies were performed on fixed brain sections to assess background staining.

Animals and other research organisms

Policy information about studies involving animals; ARRIVE guidelines recommended for reporting animal research, and Sex and Gender in Research

Laboratory animals

Strains used were either C57BL/6J male mice purchased from Jackson Labs (Bar Harbor, ME) or male LysM+/gfp offspring from C57BL/6J mice bred in our facility with LysMgfp/gfp mice. LysMgfp/gfp breeder mice were a gift from Dorian McGavern (NINDS). Mice were between 8-10 weeks at the onset of an experiment.

Wild animals

the study did not involve wild animals

Reporting on sex

While the social defeat paradigm is well-established and pharmacologically validated for the preclinical study of depression- and anxiety-like behavior, a major limitation of this and similar studies is that they are done almost exclusively in adult male mice (defined by examination of genitalia). 'Resident' CD-1 males commonly used in defeat paradigms like ours to induce depressive-like behavior do not show aggression towards 'intruder' females unless manipulated to do so, either through surgical implant (10.1038/s41598-017-12811-8) or by daily application of male odorant to females (10.1038/npp.2017.259), and unfortunately these adaptations to the standard paradigm were not published until after we had begun data collection. While alternative species like California mice exhibit territorial aggression in both sexes (10.1371/journal.pone.0017405), there are fewer reagents for examining an immune response (i.e. antibodies for flow cytometry) available and we did not have ready access to these animals.

Field-collected samples

the study did not have samples collected from the field.

Ethics oversight

All procedures were approved by the National Institute of Mental Health Animal Care and Use Committee (protocol #LCMR06) and conducted in accordance with National Institutes of Health guidelines.

Note that full information on the approval of the study protocol must also be provided in the manuscript.

Plants

Seed stocks

n/a

Novel plant genotypes

n/a

Authentication

n/a

Flow Cytometry

Plots

Confirm that:

☒ The axis labels state the marker and fluorochrome used (e.g. CD4-FITC).

☒ The axis scales are clearly visible. Include numbers along axes only for bottom left plot of group (a 'group' is an analysis of identical markers).

☒ All plots are contour plots with outliers or pseudocolor plots.

☒ A numerical value for number of cells or percentage (with statistics) is provided.

Methodology

Sample preparation	All cells were derived from C57BL/6J or LysMgfp/+ mice. Tissue collection occurred between ~8am and noon. Mice were lightly anesthetized. Venous blood was collected into EDTA tubes via puncture of the submandibular vein and kept on ice until processing. Retro-orbital injections of CD45 antibody to label blood-exposed cells were administered while mice were under light isoflurane anesthesia. After, mice were deeply anesthetized with isoflurane prior to perfusion with 35 mL room temperature PBS. For tibia bone marrow (BM), hindlegs were collected into cold HBSS + 0.1% BSA. The head was decapitated, and intact skull cleaned with a scalpel to remove muscle and connective tissue. The skull was placed in cold HBSS + 0.1% BSA on ice until further processing. ~500 µL of venous blood was lysed in 8 mL ACK Lysis Buffer (Cat. # 351-029-721; Quality Biological, Inc.) for 5 min at room temperature, and the reaction stopped by diluting with 7 mL cold HBSS + 0.1% BSA. Cells were pelleted, washed, and prepared for staining. To prepare for skull bone marrow (BM) extraction, the dorsal calvarium was trimmed to be relatively flat, and meninges were removed under a dissecting microscope. Next, the skull was cut into small bone pieces with scissors in cold HBSS + 0.1% BSA. This entire slurry was transferred to a 70 µm cell strainer and mashed with the rubber end of a 3 mL syringe for approximately 2 min per sample. Tibia were prepared by first stripping away all tissue from the bone, then cutting the very top such that a 23g syringe needle could be inserted to flush out the BM into a tube of cold HBSS + 0.1% BSA. This was next transferred to a 70 µm cell strainer and mashed with the rubber end of a 3 mL syringe. For both kinds of BM, the resulting cell suspensions were then pelleted and prepared for flow cytometry. Meninges samples were collected by first cutting around the lateral sutures of the skull; the dorsal skull and ventral skull were transferred to a fresh petri dish filled with cold HBSS + 0.1% BSA and kept on ice while pial and arachnoid meningeal membranes were gently picked off the entire outer surface of the brain into a second 'working dish' with Dumont #5 forceps (Cat. #RS-5058; Roboz, Gaithersburg, MD). Extra care was taken to avoid inclusion of choroid plexus from the 4th ventricle. Once finished with the brain, skull pieces were transferred as necessary into the working dish to remove attached meninges; we avoided leaving skull pieces in the 'working dish' to minimize contamination with cells from skull BM. Upon completion of the meningeal dissection, samples were transferred to a fresh tube and cells were pelleted by centrifugation, then resuspended in 2mL of BSA-free HBSS supplemented with 2.5 mg/mL Collagenase D (Cat. #11088858001; Roche) and 12.5 µL of 0.5 mg/mL DNAseI (Cat. #L5002139; Worthington) for cell dissociation. The samples were incubated at 37oC for 30 min, diluted with cold HBSS + 0.1% BSA, and mashed through a 70 µm cell strainer into single cell suspension.
Instrument	Data were collected on either a BD LSR Fortessa or a Beckman Coulter CytoFLEX flow analyzer
Software	Data were analyzed using FlowJo (BD) software with manual gating
Cell population abundance	Meningeal single cell suspensions were sorted into live, nucleated singlets at >98% purity.
Gating strategy	Full gating strategies are shown in supplemental figures S2a/b, S9a, S10e, S16c, S17c, S18c, S21a. S21 is presented from the point of identifying neutrophils, then IFNAR+ neutrophils; the gating strategy was identical to what is shown on the first two lines of S18C . Positive/negative gates for cell lineage were always set using blood as a guide given the well-defined expectation for population frequencies (https://www.bio-rad-antibodies.com/static/2017/flow/flow-cytometry-cell-frequency.pdf). These were adjusted slightly between tissues with different autofluorescence/debris concentration by using contour/density/and pseudocolored gates to match previously published references for myeloid cell lineages in BM (PMC4408895) and meninges (10.1038/nn.4610 and 10.1016/j.bbi.2021.08.002). For neutrophil subpopulation frequencies (CXCR2, CXCR4, IFNAR) a fluorescence minus one (FMO) control was used for each tissue type on each day of data collection to define the negative gate.

☒ Tick this box to confirm that a figure exemplifying the gating strategy is provided in the Supplementary Information.
